# Genetic structure of the grey side-gilled sea slug (*Pleurobranchaea maculata*) in coastal waters of New Zealand

**DOI:** 10.1371/journal.pone.0202197

**Published:** 2018-08-16

**Authors:** Yeşerin Yıldırım, Marti J. Anderson, Bengt Hansson, Selina Patel, Craig D. Millar, Paul B. Rainey

**Affiliations:** 1 New Zealand Institute for Advanced Study, Massey University, Auckland, New Zealand; 2 Institute of Natural and Mathematical Sciences, Massey University, Auckland, New Zealand; 3 Department of Biology, Lund University, Lund, Sweden; 4 School of Biological Sciences, University of Auckland, Auckland, New Zealand; 5 Department of Microbial Population Biology, Max Planck Institute for Evolutionary Biology, Plön, Germany; 6 Ecole Supérieure de Physique et de Chimie Industrielles de la Ville de Paris (ESPCI ParisTech), CNRS UMR 8231, PSL Research University, Paris, France; Sichuan University, CHINA

## Abstract

*Pleurobranchaea maculata* is a rarely studied species of the Heterobranchia found throughout the south and western Pacific–and recently recorded in Argentina–whose population genetic structure is unknown. Interest in the species was sparked in New Zealand following a series of dog deaths caused by ingestions of slugs containing high levels of the neurotoxin tetrodotoxin. Here we describe the genetic structure and demographic history of *P*. *maculata* populations from five principle locations in New Zealand based on extensive analyses of 12 microsatellite loci and the *COI* and *CytB* regions of mitochondrial DNA (mtDNA). Microsatellite data showed significant differentiation between northern and southern populations with population structure being associated with previously described regional variations in tetrodotoxin concentrations. However, mtDNA sequence data did not support such structure, revealing a star-shaped haplotype network with estimates of expansion time suggesting a population expansion in the Pleistocene era. Inclusion of publicly available mtDNA sequence sea slugs from Argentina did not alter the star-shaped network. We interpret our data as indicative of a single founding population that fragmented following geographical changes that brought about the present day north-south divide in New Zealand waters. Lack of evidence of cryptic species supports data indicating that differences in toxicity of individuals among regions are a consequence of differences in diet.

## Introduction

The grey side-gilled sea slug *(Pleurobranchaea maculata)* is an opportunistic carnivore that feeds on invertebrates including sea anemones, marine worms and other molluscs [1] but also on algae [2]. It is native to New Zealand (NZ), southeastern Australia, China, Sri Lanka and Japan where it is found in habitats ranging from sandy sediments to rocky reefs, and from shallow sub-tidal flats to depths of 300 m [1, 3]. Little is known about the life history of the species but studies of comparative development report the production of planktotrophic veligers that hatch within eight days and remain planktonic for three weeks before juveniles settle [1, 3, 4].

In late 2009 this otherwise little-known sea slug attracted attention after it was implicated in dog deaths on beaches in Auckland [5]. Analyses of vomit and gastrointestinal contents revealed that deaths were a consequence of tetrodotoxin (TTX) poisoning associated with ingestion of *P*. *maculata* [5]. This was the first time that TTX had been reported in NZ and in a species of the taxonomic clade Heterobranchia [5]. *P*. *maculata* that have recently invaded coastal waters of Argentina also contain TTX [6, 7].

TTX is a potent neurotoxin found in numerous terrestrial and marine organisms, but neither the origin of TTX nor the causes of variation in TTX levels among species are understood. The structure of TTX suggests a microbial origin [8] and while certain microbes have been implicated in TTX production (reviewed in [9]), many of the claims have been refuted [9, 10]. Nonetheless, while not excluding a microbial origin, there is recognition that TTX in animals is often acquired via diet. For example, variability in TTX levels found in puffer fish has been attributed to exposure to toxic food sources (reviewed in [8]). For *P*. *maculata*, there is mounting evidence that toxin accumulation occurs through feeding [11–14]. An alternate possibility is that TTX arises from commensal or symbiotic microorganisms that are associated with *P*. *maculata* [14], but no TTX-producing bacteria have been found [15, 16]. Observation of a significant number of egg masses during the period when toxin levels peak in TTX-associated *P*. *maculata* populations [3], and vertical transfer of TTX from adults to [13] suggests that TTX may serve a defensive function.

Studies of individual and temporal differences in TTX concentration have established that *P*. *maculata* populations from northern regions of the North Island (Whangarei, Auckland, Tauranga) have high TTX concentrations (the highest average being 368.7 mg kg^-1^ per individual), while populations from the South Island (Nelson and Kaikoura) have trace amounts of TTX (<0.5 mg kg^-1^) or none at all [3, 5, 11, 14]. A recent study reported TTX concentrations as high as 487 mg kg^-1^ [14]. Significant individual and seasonal variations have also been observed [3]. A single individual obtained from Wellington in the south of the North Island was found to have a low concentration of TTX (2.2 mg kg^-1^) supporting the notion of a geographical cline [3].

The genetic structure of *P*. *maculata* is unknown, but variation in the established differences in toxicity between northern and southern populations suggests that geographic variability in TTX concentration correlates with genetic structure–even the possibility that northern and southern populations define separate species. Here we test this hypothesis using a combination of microsatellite and mitochondrial DNA (mtDNA) sequence markers.

## Materials and methods

### Sampling, DNA extraction and tetrodotoxin assay

**Fig 1:**
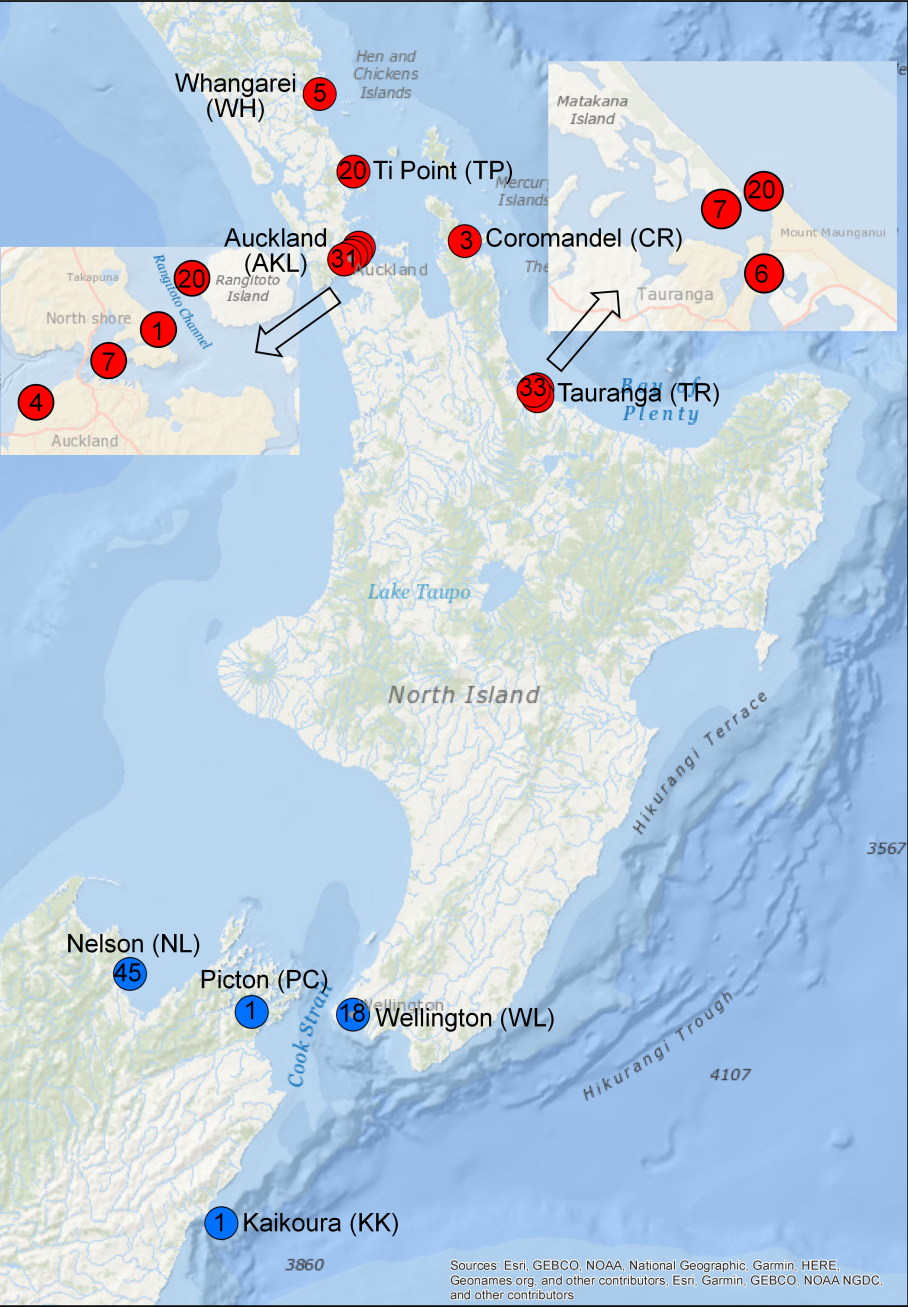
Sampling locations for the *Pleurobranchaea maculata* individuals. The numbers within the circles indicate the sampling size of each region. The arrows show magnified maps of Auckland and Tauranga. Populations containing *P*. *maculata* individuals with high, and low and trace amounts of tetrodotoxin concentrations in red and blue colour, respectively.

A total of 156 samples were collected from nine regions around New Zealand between 2009 and 2013 (Fig 1 and Table A in S1 File). DNA was extracted as described in Yıldırım *et al*. [17]. The Tauranga (TR) population included samples from Tauranga Harbour whereas the Auckland (AKL) population included samples from Tamaki Strait and Waitemata Harbour. Some samples were from the studies of Wood *et al*. [3] and Khor *et al*. [11].

At the outset of this study there was limited knowledge of the toxicity of *P*. *maculata* individuals from Wellington (WL) as only one individual had been previously tested [3]. To obtain a better understanding, the TTX concentration of eight (of eighteen) individuals collected from WL in October 2012 was determined as described in Khor et al. [11]. The TTX assay was performed at the Cawthron Institute (Nelson) using a liquid chromatography-mass spectrophotometry method that is described in McNabb et al. [5].

Population-level analyses were performed only for five populations which are Ti Point (TP), AKL, TR, WL and Nelson (NL) due to the small sample sizes of the other locations. TP, AKL and TR, which included highly toxic individuals [11] were designated as the “northern cluster”, whereas the WL and NL population, which contained slightly toxic and non-toxic individuals [3, 11, 12] were designated as the “southern cluster”.

### Genotyping

Twelve microsatellite markers (*Pm01*, *02*, *07*, *08*, *09*, *10*, *11*, *13*, *17*, *19*, *20* and *23*) [17] were genotyped for 149 samples. PCR amplification and genotyping procedures for the primers were as described in Yıldırım *et al*. [17] with some modifications (Table B in S1 File). Details regarding amplification and genotyping processes are described in the Supporting Information.

A 1060 bp and 1153 bp region of mitochondrial cytochrome B (*CytB*) and cytochrome oxidase I (*COI*) genes, respectively, were amplified and sequenced in all 156 *P*. *maculata* individuals. For details regarding the primer pairs and amplification see Methodology and Table C in S1 File. Geneious Pro 6.1.6 (Biomatters, New Zealand) was used to trim, assemble, align and concatenate the resulting DNA sequences.

### Statistical analysis

#### Genetic diversity

Microsatellite genotyping data were tested for scoring errors due to stuttering, null alleles, and large allele dropout using MICRO-CHECKER v.2.2 [18]. Departures from Hardy-Weinberg equilibrium (HWE) were estimated using inbreeding coefficient *F*_*IS*_ [19] with 9,999 permutations in GenoDive v2.0b25 [20]. Correction for multiple testing was performed using the false discovery rate (FDR) method [21]. Linkage disequilibrium (LD) between pairs of loci was estimated in FSTAT v2.9.3.2 [22] for each population and across populations. The significance of LD was determined by applying a Bonferroni correction using 6,600 permutations for a 5% nominal level. The total number of alleles (*Na*), allele frequencies, observed heterozygosity (*H*_*o*_), expected heterozygosity (*H*_*e*_) [23–25], and private alleles (*PA*) per locus and population were calculated using GenAlex v6 [26]. Allelic richness (*A*_*R*_) and private allelic richness (*PA*_*R*_) were calculated using the rarefaction method implemented in ADZE v.1.0 [27]. *H*_*e*_ and *A*_*R*_ were used to compare the amount of genetic diversity among populations from different regions using one-way Wilcoxon-Mann-Whitney Test [28].

For mtDNA, several estimates of genetic diversity, including the number of singletons (*Sin*), haplotypes (*Hap*) and segregating sites (*S*), the average number of nucleotide differences between sequences (*k*) [29], haplotype (*h*) and nucleotide diversity (*π*) [30] were calculated for the *CytB* and *COI* for each sampling location using DnaSP 5.10.0.1 [31].

#### Population structure

For microsatellite data, global differentiation and pairwise differentiation between each pair of populations was investigated using various differentiation estimators, including a log-likelihood ratio (G)-based test [32], fixation index *F*_*ST*_ [19], and Jost’s [33] differentiation (*Dest*) in GenAlex. The statistical power to detect true population differentiation and α-error probability were assessed in POWSIM v4.1 [34]. STRUCTURE v2.3.4 [35] was used to determine the probable number of distinct populations (*K*) and individuals were assigned to populations using a Bayesian assignment approach. The most likely value of *K* was resolved using the ΔK method [36] with the Structure Harvester v0.6.93 [37], and the results were introduced to the CLUMPP v1.1.2 software [38]. Destruct v1.1 [39] was used to visualize the results (see the Supporting Information for used parameters for POWSIM and STRUCTURE).

For the mtDNA sequences, POPART v1 (http://www.leigh.net.nz/software.shtml) was used to create a median joining haplotype network (MJN) [40]. We created an additional MJN for shorter COI sequences (624 bp) in order to accommodate four *P*. *maculata* samples obtained from individuals isolated in Argentina (Farias et al., 2016). A saturation test was performed in DAMBE v6.2.9 [41] using the test by Xia et al. [42]. The proportion of invariant sites (*P*_*in*v_) was estimated by Jmodeltest v0.1.1 [43] with the Akaike information criteria (AIC). The *P*_*inv*_ values (0.844 and 0.789 for *CytB* and *COI*, respectively) obtained from the most likely models suggested by the software (HKY+I and TrN+I for *CytB* and *COI*, respectively) and default settings for other parameters were used for the calculations. Haplotype-frequency-based *F*_*ST*_ [44] and distance-based *Θ*_*ST*_ [45] were calculated in ARLEQUIN v3.5 [46] to estimate population differentiation. For *Θ*_*ST*_, the Tamura-Nei mutational model [47] was used for both genes as being the closest models to the ones suggested as most likely to explain mtDNA data by Jmodeltest.

Patterns of differentiation were also analysed using a multivariate approach. For microsatellite data, the Manhattan distance (*DM*), which calculates the mean character differences between individuals, and clonal distances (*DCL*), which assumes a stepwise mutational model (SMM) [45], were used. For mtDNA data, a distance matrix (*D*_*SEQ*_) was calculated as a standardized bp difference between every pair of individuals in Mothur v1.33.3 [48]. Statistical analyses on resulting distance matrices were done using PRIMER v6 [49] with PERMANOVA+ [50]. Patterns of inter-sample distances were visualized using non-metric multi-dimensional scaling ordination (MDS) [51]. Permutational multivariate analysis of variance (PERMANOVA) [52, 53] was used to formally test for differences in genetic structures among different locations and canonical analysis of principal coordinates (CAP) [50, 54] was used to discriminate among specific populations and identify their distinctiveness, using leave-one-out allocation success. PERMDISP was used to test the null hypothesis of homogeneity of within-group dispersions among populations [55]. All permutation tests used 10,000 permutations. Multivariate analyses were used as an alternative approach because they do not require strong assumptions about an underlying genetic model of population structure. The method was also used to investigate the relevance of north-south disjunction for both microsatellite and mtDNA data.

A maximum likelihood (ML) tree using the Tamura-Nei mutational model [29] with default settings was reconstructed for 44 *P*. *maculata* individuals from this study and three *P*. *maculata* individuals from Argentina using COI sequences [6] (redundant sequences were removed) in MEGA7 [56]. COI sequences of five species from the same family (Pleurobranchidae, Genbank codes in brackets) including *Pleurobranchaea meckeli* (KU365727.1), *Pleurobranchaea nevaezelandiae*, *Pleurobranchus peronii* (KM521745.1), *Berthella ocellata* (KM521694.1) and *Berthellina citrina* (KM521694.1) were used as out-groups. The analysis involved 200 informative positions of 616. The phylogeny was tested with 1,000 bootstrap replicates.

#### Migration

The microsatellite data were analysed with GeneClass2 [57] to identify the first-generation migrants using the Bayesian criterion of Rannala and Mountain [58] and the L_home_/L_max_ likelihood by introducing northern and southern clusters as populations, with a threshold *p-*value of 0.01 and a Monte-Carlo resampling algorithm [59].

#### Neutrality tests and demographic analyses

BOTTLENECK v1.2 [60] was used to test the possibility of recent population reduction for microsatellite data assuming SMM and two-phase models (TPM) with default settings using a Wilcoxon signed rank test [60]. A possible sign of a recent bottleneck was investigated also by a mode-shift analysis [61].

We used isolation-with-migration models [62] implemented in the program IMa2, [63] to evaluate historical demographic parameters of the two main *P*. *maculata* populations (as determined by the phylogenetic reconstructions), i.e., northern and southern populations, using the microsatellite allele data (twelve loci). IMa2 uses Bayesian inference and MCMC simulations of genealogies to estimate several demographic parameters, including population size (*Θ*) of the extant (*Θ*_North_ and *Θ*_South_) and ancestral populations (*Θ*_Ancestral_), the split time (*t*) for the branching event, and asymmetric migration rates between the extant populations (*m*_*North→South*_, *m*_*South→North*_). A step-wise mutation model was applied. Upper bounds of prior distributions of parameter values were evaluated in several trial runs. When the highest posterior probability peaks of all parameters fell well within the prior boundaries in these test runs, we ran five IMa2 runs with prior settings chosen according to these trial runs (three with wider and two with narrower prior boundary settings), all with different random seed numbers. The runs began with a burn-in period of 10^6^ steps followed by 10^8^ steps where every 10^3^ genealogy was sampled. We achieved adequate convergence and mixing of the Markov chains as indicated by visual inspection of trend line plots, sufficient effective sample size values and similar posterior probability distributions in the five runs. We present parameter estimates corresponding to the average highest peak of the posterior probability distribution of the five runs. The parameter estimates are scaled to the mutation rate (*μ*) and generation time (*g*) and to convert them to biologically interpretable demographic units, we calculated the population sizes (*N*) as *Θ*/4*μ*, split time (*T*) in years as *tg*/*μ*, and population migration rates per generation (*2NM*) as *Θm/2*. The mutation rate is uncertain for *P*. *maculata* microsatellites and was set to 10^−4^ per generation, a commonly assumed mutations rate for microsatellites [64, 65], and the generation time was set to one year. Note that the direction of gene flow for *2NM* is forward in time (e.g., *2Θ*_*North*_*m*_*South→North*_ indicates the number of migrants per generation that the northern population receives from the southern population).

Deviations from neutrality and demographic changes within and across the populations were calculated with Tajima’s *D* [66], Fu’s *Fs* [67] and mismatch distribution analysis in ARLEQUIN for the mtDNA *COI* and *Cytb* sequences. The null hypothesis of expansion was statistically tested with the sum of squared deviations (*SSD*) from the expected values [68] and Harpending’s *raggedness* index [69]. McDonald and Kreitman’s [70] neutrality test was performed pooling all *P*. *maculata* COI sequences (1153 bp) in DnaSP using *P*. *meckeli* COI sequences as an outgroup species. Fisher's exact test (two-tailed) was used to identify significant deviations from neutrality.

Population size reconstruction based on the *COI* and *CytB* sequence data using Bayesian skyline plot analyses [71] was performed in BEAST 2.4.3 [72]. Since there was no evidence of mtDNA population structure (see Results) a single population was modelled. The input was prepared in BEAUti (part of BEAST suite). We used the HKY substitution model with five gamma categories, an exponential gamma shape prior of 1.0 and a log-normal kappa of 10.0, and a strict clock model with a uniform clock rate prior (parameters estimated empirically). The substitution rate was set to 2.0%/My (the average substitution rate used in several recent studies of marine invertebrates [65, 73]. In the MCMC, a chain length of 1.1 × 10^8^ and a pre-burn-in of 10^7^, with sampling every 10^4^, were used. Results were inspected and the Bayesian skyline plot analysed and reconstructed in Tracer 1.7 [74].

## Results

### Tetrodotoxin levels in *P*. *maculata* from Wellington

Previous analyses have established that northern WH, AKL, TR, and Coromandel populations have high levels of TTX, marking these populations as “toxic”, while southern populations from NL and Kaikoura (KK) are recorded as containing either trace, or no TTX [3, 5, 11, 14]. For WL populations, previous measurements existed for only one individual documented as having a low level of TTX (2.2 mg/kg) [3]. For this study, 18 slugs were obtained from WL of which eight randomly chosen individuals were subject to TTX assay. Three contained extremely low concentrations (0.12, 0.16 and 0.5 mg/kg) of TTX. No TTX was detected in the remaining five individuals. Accordingly, the WL, NL and KK samples (the southern cluster) were classified as “non-toxic”.

### Genetic diversity

#### Microsatellite analyses

All loci were highly polymorphic with between five and 23 alleles for each locus (diversity statistics in Table 1 and Table D in S1 File). *H*_*e*_ across populations ranged from 0.407 to 0.843, with an average of 0.655. Rarefaction curves for *A*_*R*_ across each locus levelled off for each sampling location indicating that a reasonable portion of the available allelic diversity was sampled at each location (Figure A and in Table E in S1 File). Populations did not exhibit significant differences in genetic diversity for either *A*_*R*_ (*P*≥0.370) or *H*_*e*_ (*P*≥0.504). No significant linkage disequilibrium was found after Bonferroni correction (*P*>0.05) (Table F in S1 File). Populations met Hardy-Weinberg expectations (MICROCHECKER, Table 1) after FDR correction (Table D in S1 File).

**Table 1:** Summary of the genetic diversity statistics at microsatellite loci across five locations.

Locus	Na	Size (bp)	*H*_*O*_	*H*_*e*_	*F*_*IS*_	*F*_*ST*_	*Dest*
*Pm01*	23	108–208	0.842	0.826	-0.020	0.057^b^	0.291^b^
*Pm02*	9	105–137	0.671	0.826	0.111	0.026	0.033
*Pm07*	10	128–164	0.737	0.826	-0.059	0.014	-0.005
*Pm08*	6	141–165	0.710	0.826	-0.152	0.115^b^	0.274^b^
*Pm09*	16	91–142	0.733	0.826	-0.026	0.045^b^	0.108^b^
*Pm10*	6	107–122	0.737	0.826	-0.057	0.071^b^	0.175^b^
*Pm11*	11	157–187	0.838	0.826	-0.062	0.035^a^	0.114^a^
*Pm13*	8	103–136	0.572	0.826	-0.082	0.007	-0.014
*Pm17*	8	155–187	0.457	0.826	-0.029	0.058^a^	0.046^a^
*Pm19*	5	169–181	0.523	0.826	-0.039	0.041^a^	0.036^a^
*Pm20*	5	114–132	0.407	0.826	0.016	0.181^b^	0.174^b^
*Pm23*	14	156–184	0.703	0.826	-0.007	0.132^b^	0.378^b^
Ave	6.667		0.661	0.642	-0.034	0.064^b^	0.122^b^
SE	0.428		0.020	0.018	0.018	0.014	0.035

Significant genetic differentiation

^a^ (*P*<0.05)

^b^ (*P*<0.001).

#### Mitochondrial DNA analyses

The basic diversity values for *COI* and *CytB* sequences are presented in Table 2. The total number of variable sites is 173 (*COI*: 105; *CytB*: 68), 98 of which are singleton mutations (*COI*: 59; *CytB*: 39); which defined 103 and 74 distinct haplotypes for *COI* and *CytB* sequences, respectively. In contrast to the range of values obtained for *h* (0.922–0.989), the corresponding ranges for *k* (2.9–4.8) and π (0.254–0.414%) were low for both genes. The mean values for *k* and *π* are 7.30 (*COI*: 3.81±0.018, *CytB*: 2.75±0.018) and 0.330% (*COI*: 0.381%, *CytB*: 0.275%), respectively for the concatenated sequences. Similar diversity was observed between sampling locations.

**Table 2:** Summary of the genetic diversity statistics for individuals sampled from five locations.

		Microsatellite					*COI*						*CytB*					
Pop	*N*	*Na*	*A*_*R*_	*H*_*O*_	*H*_*e*_	*F*_*IS*_	*S*	*Sin*	*Hap*	*h±*SD	*k*	*π*±SD%	*S*	*Sin*	*Hap*	*h±*SD	*k*	*π*±SD%
Ti Point	20	6.08	6.02	0.679	0.663	0.001	27	16	17	0.984±0.020	4.8	0.414±0.048	17	9	16	0.957±0.021	3.1	0.294±0.041
Auckland	30	6.67	5.88	0.681	0.656	-0.021	39	31	26	0.989±0.013	4.1	0.352±0.033	23	15	18	0.922±0.034	2.7	0.254±0.043
Tauranga	33	6.88	5.89	0.697	0.665	-0.032	39	27	27	0.981±0.015	4.5	0.390±0.045	29	20	21	0.936±0.032	2.8	0.263±0.041
Wellington	18	6.00	6.00	0.606	0.618	0.047	25	17	16	0.987±0.023	4.2	0.363±0.033	18	10	13	0.954±0.034	3.4	0.317±0.046
Nelson	45	7.75	5.93	0.641	0.609	-0.041	44	33	30	0.957±0.018	4.0	0.348±0.035	35	23	29	0.941±0.063	2.9	0.276±0.032
Total	146	121	-	0.661	0.642	-0.009	105	59	103	0.980±0.005	4.4	0.381±0.018	68	39	74	0.945±0.011	2.9	0.275±0.018

*N*: sample size, *Na* number of alleles, *A*_*R*_: mean allelic richness based on 18 diploid individuals, *Ho* observed heterozygosity, *He*: expected heterozygosity, *F*_*IS*_: inbreeding coefficient. *S*: number of segregating sites, *Sin*: singleton mutations, *Hap*: number of haplotypes detected, *h*: haplotype diversity, *k*: number of pairwise nucleotide differences, *π*: nucleotide diversity, SD: standard deviation.

### Genetic structure

#### Microsatellite analyses

Global genetic differentiation, estimated using *F*_*ST*_ and *Dest*, was low to moderate: 0.064 and 0.122, respectively, and highly significant (*P*≤0.0001). Differentiation was significant for most loci (Table 1). Pairwise comparisons with all estimators (Genic: *χ2* = infinity, d.f. = 24; *F*_*ST*_ = 0.074–0.122; and *Dest* = 0.153–0.246) showed that southern populations (WL and NL) were significantly differentiated from northern populations (TP, AKL and TR) (*P*<0.0001, Table 3 and Table G in S1 File). Most loci supported this pairwise differentiation pattern among populations (*Dest* values in Table H in S1 File). None of the estimators suggested significant differentiation among the northern populations of TP, AKL and TR. A weak but significant differentiation between the WL and NL populations (*P*<0.046) was found based on some estimators (Table G in S1 File). Analysis of statistical power by POWSIM showed a 100% probability of detecting population differentiation at an *F*_*ST*_ value of 0.01. The probability of α error was ~0.05 (*P* = 0.04 and 0.057 based on chi-square and Fisher methods, respectively), suggesting a low risk for Type I error. The differentiation pattern was therefore considered real and reinforced by *F*_*ST*_ values between the significantly differentiated populations being for the most part greater than 0.01.

**Table 3:** Pairwise population differentiation estimates and associated tests across five populations.

	Microsatellite data			*COI* data		*CytB* data*	
Groups	Genic	*F*_*ST*_	*Dest*	*F*_*ST*_	*Θ*_*ST*_	*F*_*ST*_	*Θ*_*ST*_
TP-AKL	17.26	-0.009	-0.019	0.014^b^	0.024	0.050^c^	0.028
TP-TR	29.67	-0.004	-0.008	0.017^b^	0.018	0.043^b^	0.023
TP-WL	Inf^c^	0.087^c^	0.185^c^	0.014^a^	0.008	0.033^c^	-0.026
TP-NL	Inf^c^	0.118^c^	0.241^c^	0.030 ^b^	0.029	0.040 ^b^	0.000
AKL-TR	20.48	-0.004	-0.007	0.015^c^	0.020	0.071^c^	0.019
AKL-WL	Inf^c^	0.095^c^	0.198^c^	0.012^a^	0.014	0.062^c^	-0.003
AKL-NL	Inf^c^	0.122^c^	0.246^c^	0.028^c^	0.005	0.068^c^	-0.001
TR-WL	Inf^c^	0.074^c^	0.153^c^	0.016^a^	0.017	0.056^c^	0.012
TR-NL	Inf^c^	0.097^c^	0.194^c^	0.031^c^	0.007	0.061^c^	0.026^a^
WL-NL	53.20^b^	0.008^a^	0.013	0.029^b^	0.020	0.053^c^	-0.014

*χ2* values for the homogeneity of allele frequencies in pairwise comparisons tested with the exact *G*-test (Genic). Other estimators used were fixation index *F*_*ST*,_ Jost’s differentiation (*Dest*) and distance-based *Θ*_*ST*._

TP: Ti Point, AKL: Auckland, TR: Tauranga, WL: Wellington, NL: Nelson. Significant differentiation

^a^ *P*<0.05

^b^ *P*<0.0005

^c^ *P*≤0.0001.

**Fig 2:**
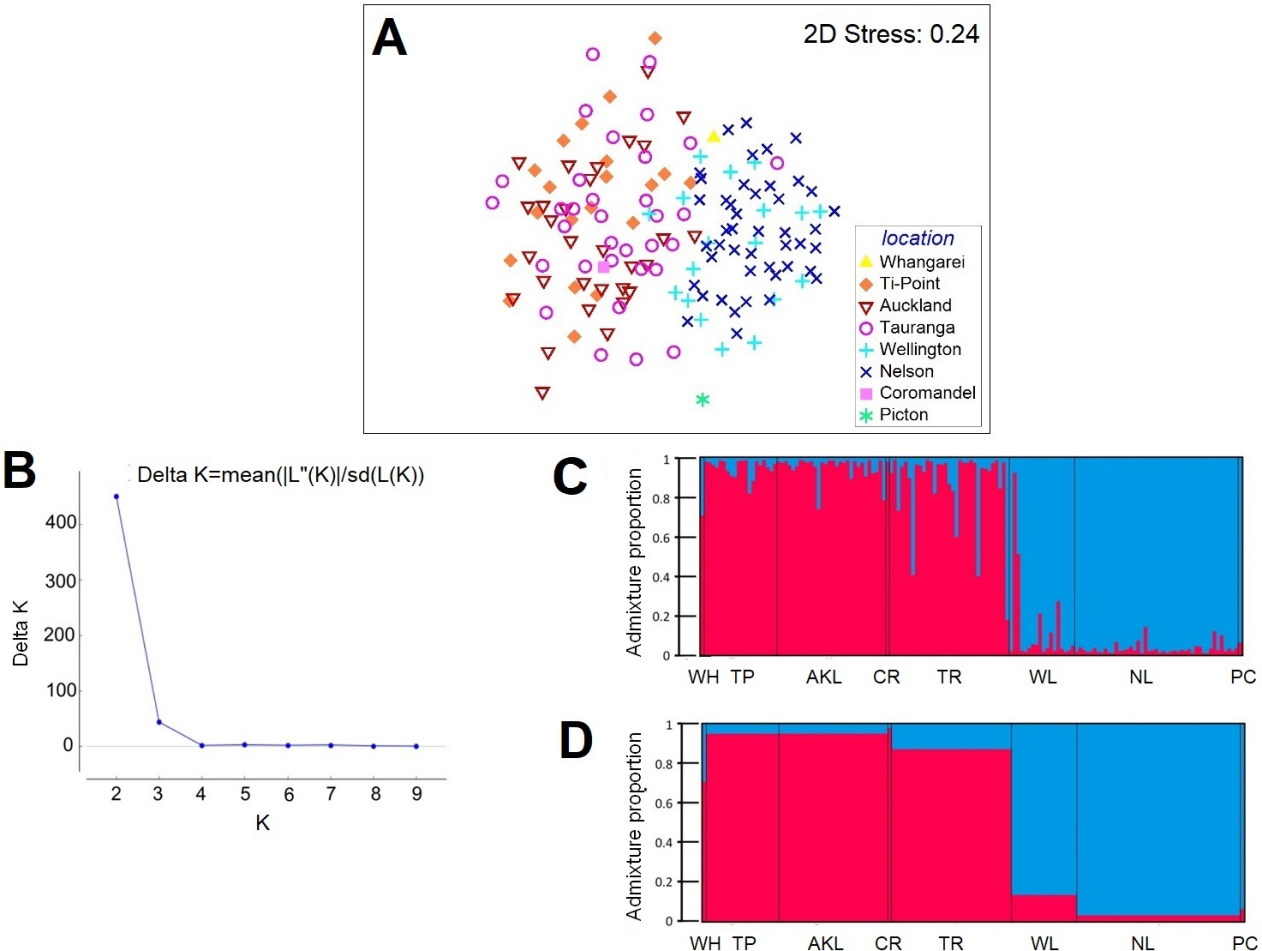
Visualization of genetic structure among *Pleurobranchaea maculata* populations based on microsatellite data. All individuals from eight sampling locations were included. MDS ordination of pairwise (A) Manhattan (*DM*) distances between individuals. Bayesian clustering analysis where the sampling location was not introduced for the calculations, (B) Plot of *ΔK* versus *K* indicating that data are best explained by *K* = 2 clusters, (C) Population structure at *K* = 2. Each individual is represented by a vertical line divided into two segments, which indicates proportional membership in the two clusters; (D) Group assignments, indicating proportional membership in *K* = 2 clusters.

A north-south differentiation is evident from MDS ordinations of the *DM* (Fig 2A) and *DCL* (not shown). Note that although stress is relatively high (0.23) for the 2-dimensional MDS ordinations, the north-south distinction was also very clearly apparent in the corresponding 3-dimensional MDS ordinations (not shown here), which had lower stress (0.18). Additionally, PERMDISP analysis showed no significant differences in dispersion for either *DM* (*F*_4,141_ = 0.1243, *P* = 0.1243) or *DCL* (*F*_4,141_ = 0.4856, *P* = 0.4856), meaning that average nucleotide distances from individuals to their own population centroid did not differ among the groups (i.e. that within-group genetic variability did not differ among populations). Discriminant analysis with CAP supported (Figure B in S1 File) the two-group north-south split (*P*<0.001), with the CAP model showing a leave-one-out allocation success of 97.26% (142/146 for *DCL* and also for *DM*), while there was no discrimination among the three northern (TP, AKL and TR) and two southern populations (WL and NL) (CAP, *P*>0.76 for all matrices), justifying their pooling into a single group. Bayesian clustering of individuals based on allele frequencies as implemented by STRUCTURE showed a Δ*K* value and mean log probabilities of data (Ln*P* (x/*K*)) that were maximal at *K* = 2 (Fig 2B), again supporting the same two distinct north-south clusters (Fig 2C and 2D). This finding was not affected by inclusion of sampling locations as priors (data not shown).

#### Mitochondrial DNA analyses

**Fig 3:**
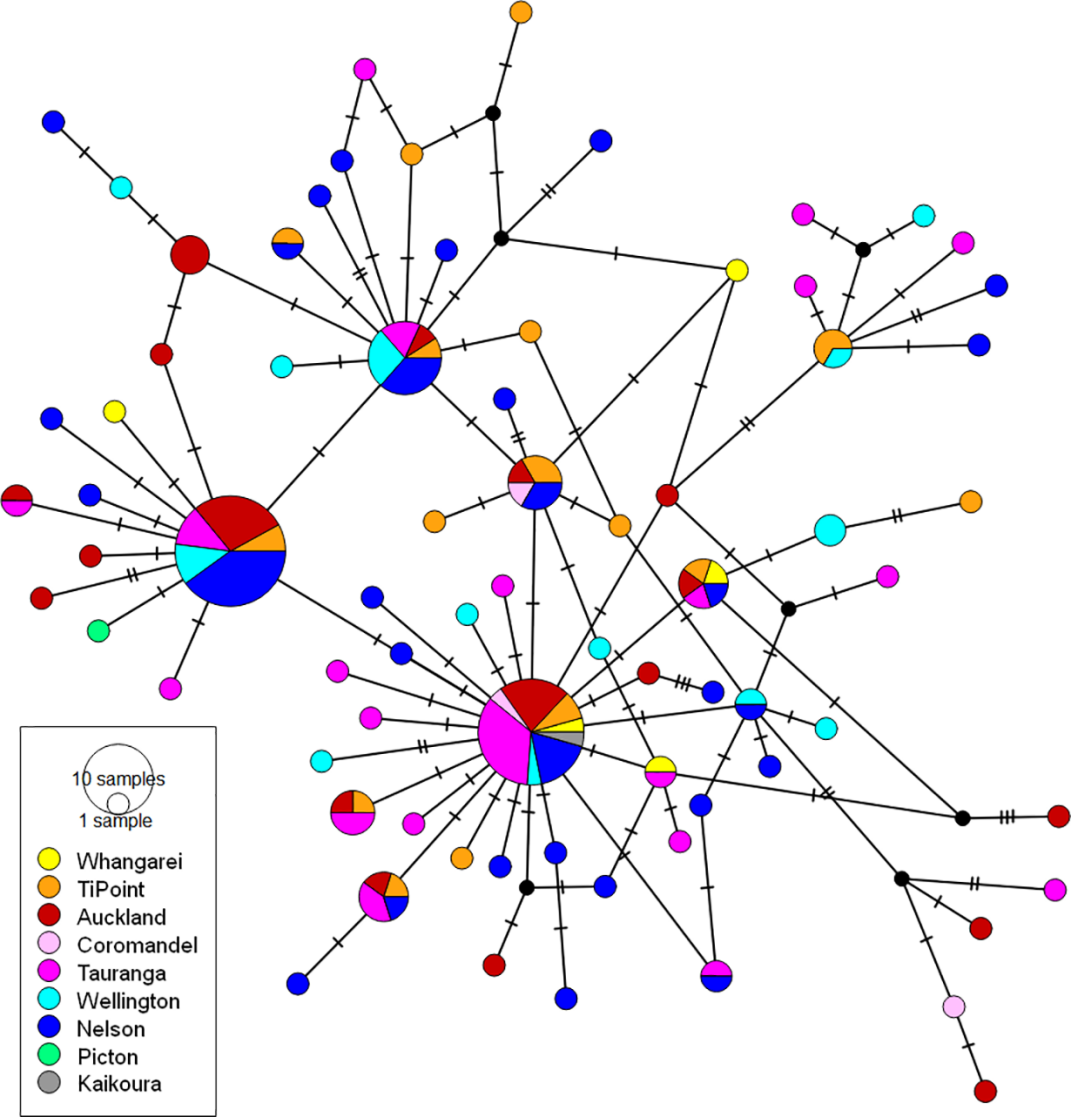
Median joining network of the *CytB* haplotypes. The network is coloured according to the sampling locations. The diameter of the circles reflects relative haplotype frequencies. The hashes indicate the mutational steps between the haplotypes. The black nodes represent the unsampled haplotypes necessary to create a bridge between the present haplotypes.

The sequence data for 156 individuals obtained from *COI* and *CytB* were submitted to GenBank (accession numbers: KY094153—KY094309 for *COI* and KY094310—KY094466 for *CytB*). MJN analysis of *CytB* (Fig 3) and *COI* (Figure C in S1 File) sequences resulted in highly similar patterns. Thus, for simplicity, the *CytB* network was used to infer evolutionary relationships among individuals. The network shows a lack of noticeable geographical structure; common haplotypes are shared across populations. The two most common *CytB* haplotypes are shared by 25 (16.0% of the total dataset) and 22 (14.1% of the total dataset) individuals (frequencies in Table I in S1 File). The network is complex and reticulated with a star-like topology; many private haplotypes descend from central shared nodes with mostly one to two base pair distances [75]. Private haplotypes were detected in all sampling locations. Characteristics of the network showed little change on inclusion of four recently published samples from Argentina (Farias *et al*, 2015; Farias *et al*, 2016) (based on a slightly shorter *COI* fragment (see Materials and Methods and Figure C in S1 File). Samples from Argentina are closely related to NZ samples with just a single base pair distance from a commonly shared haplotype. The index of substitution saturation (ISS) was used to test for homoplasy due to multiple substitutions [42]. For both symmetrical and asymmetrical tree topology models and for both genes, the observed ISS values are significantly larger than the critical *ISS* (*ISS*.*c*) values (Table J in S1 File), which indicate that the paired partitions are not saturated, and the degree of homoplasy is low.

**Fig 4:**
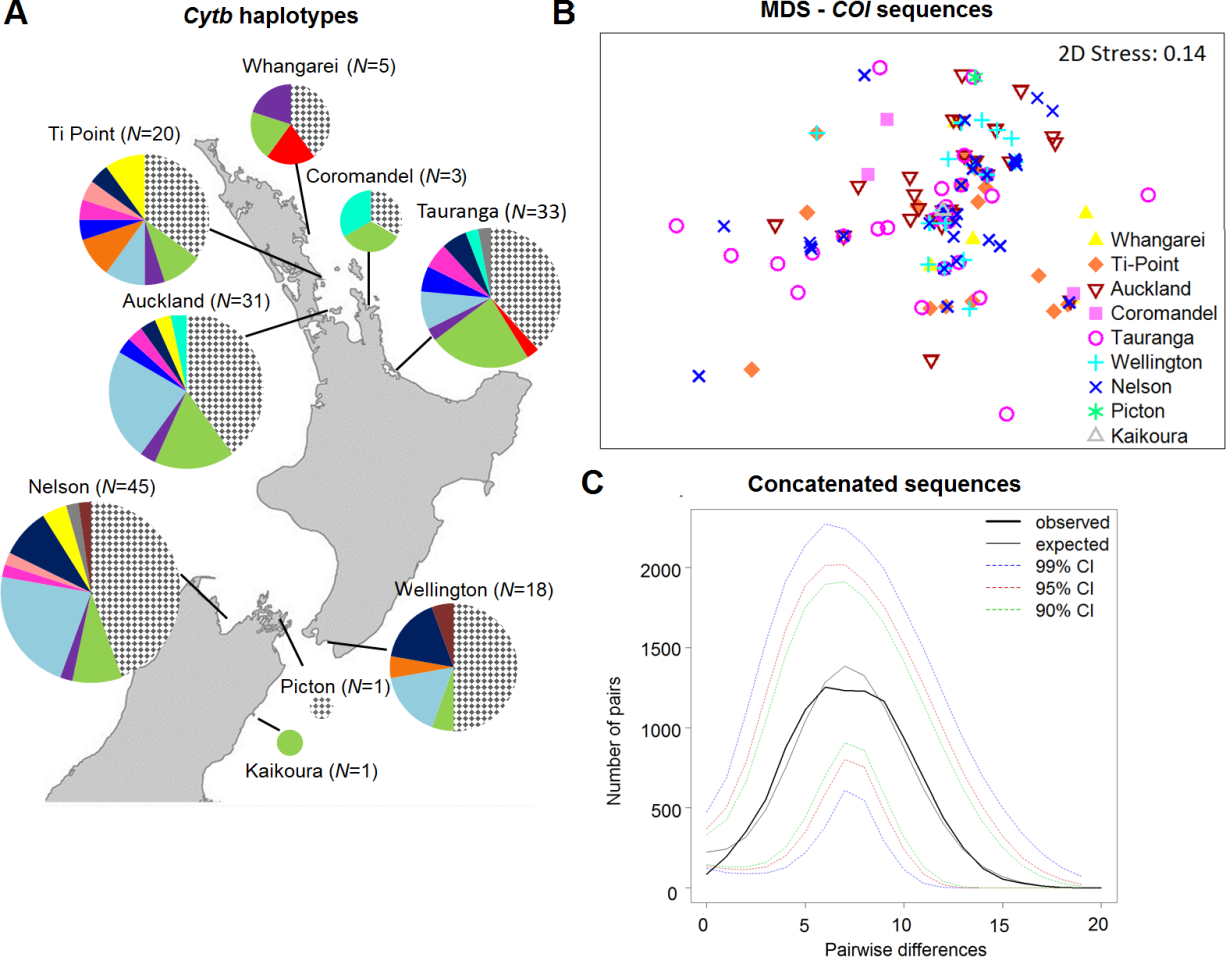
Graphical visualization of the results of population structure and demographic analysis for mtDNA data. (A) The frequencies of *CytB* haplotypes at each location (*N* = 156). The pie segment represents the relative haplotype frequencies. Each colour corresponds to a different haplotype. The patterned segment represents private haplotypes. The sizes of the circles are proportional to the sample size. (B) Non-metric MDS ordination of distances obtained from the standard nucleotide differences between individuals for the *COI* data. Each symbol also represents a different sampling location. (C) Mismatch distributions of pairwise base pair differences between the concatenated *COI* and *CytB* haplotypes.

Analysis based on *F*_*ST*_ values showed a moderate but highly significant global genetic differentiation for both *COI* (*F*_*ST*_ = 0.022, *P* = 0.0001) and *CytB* (*F*_*ST*_ = 0.057, *P* = 0.0001). However *Φ*_*ST*_ values were significant for *COI* (*Φ*_*ST*_ = 0.01552, *P* = 0.022), but not for *CytB* (*Φ*_*ST*_ = 0.00845, *P* = 0.12). Pairwise *F*_*ST*_ values were low (0.033–0.074), yet significant for all the comparisons (*P*< 0.03) (Table 3). Differences in the distribution of allele frequencies were observable for both *CytB* (Fig 4A) and *COI* (Figure D in S1 File) and pairwise *Θ*_*ST*_ values were low for all the comparisons for both *COI* and *CytB* (0.007–0.029); a weak significant differentiation was observed only between TR and NL for *CytB* (*Θ*_*ST*_ = 0.026, *P* = 0.0226, Table 3). MDS ordination of the *D*_*SEQ*_ matrices revealed no observable structure for either gene (Fig 4B). However, PERMANOVA analysis suggests that sampling location has a significant effect on the population structure for *COI* (*F*_4, 145_ = 1.8791, *P* = 0.0173), but not for *CytB* (*F*_4, 145_ = 1.3386, *P* = 0.1806), which was consistent with the results of *Φ*_*ST*_ analysis. Pairwise PERMANOVA tests showed significant but weak differentiation between the AKL and TP (*P* = 0.0276), and AKL and TR populations (*P* = 0.0218) (Table G in S1 File). PERMDISP revealed no significant differences in dispersion among populations for either *COI* (*F*_4,141_ = 0.3793, *P* = 0.847) or *CytB* (*F*_4, 141_ = 0.358, *P* = 0.833). The CAP results were consistent with the structures revealed by PERMANOVA: among the five populations, AKL is weakly distant from TP and TR based on *COI* data.

PERMANOVA analysis was repeated by contrasting the TP, AKL and TR populations (the northern population cluster) against WL and NL (the southern population cluster) to test whether the north-south differentiation identified with microsatellite data could be observed with the mtDNA data. The results indicate no significant differentiation between the clusters (*COI*: *F*_1, 145_ = 0.819, *P* = 0.5268; *CytB*: *F*_1, 145_ = 1.2325, *P* = 0.2982, Figure E in S1 File) and do not support the north-south differentiation identified by microsatellite data.

A phylogenetic analysis of *P*. *maculata* shows that *P*. *maculata* samples from NZ form a single clade (Figure F in S1 File). Inclusion of the sequences from Argentina did not change the topology of the tree. Phylogenetic relationships between *P*. *maculata* individuals were unsupported (bootstrap values <50%), whereas there was good bootstrap support (between 74–100%) for five other species in the Pleurobranchidae family.

### Migration and demographic changes

#### Microsatellite analyses

Migration analysis with GeneClass2 detected four first generation migrants (*P* = 0.01): two individuals sampled from the northern cluster (TR) were migrants from the southern cluster, and two individuals sampled from the southern cluster (WL) are migrants from the northern cluster (Table K in S1 File). Individuals from each cluster were more likely to belong to populations from the same cluster. However, presence of some first generation migrants between the clusters shows that these clusters are genetically connected. This was compatible with the results of CAP and STRUCTURE showing low connectivity between the clusters and admixed individuals in both northern and southern clusters (Fig 2C and 2D). The highest misclassifications between the clusters detected by CAP analysis and highest admixture proportions detected by STRUCTURE were noted in TR and WL. This suggests that the TR and WL populations could be bridges between the northern and southern clusters. Admixture in the WL population may also explain weak differentiation between the WL and NL populations that was found with *F*_*ST*_ and PERMANOVA tests.

The Wilcoxon test did not detect recent bottlenecks in any population under either TPM or SMM models (Table 4). In addition, analysis of mode-shift in the distribution of allele frequencies suggests that all the populations exhibit a normal L-shaped pattern indicating no mode-shift in the frequency distribution of alleles. Taken together these data suggest that none of the populations experienced a recent or sudden bottleneck.

**Table 4:** Results of the neutrality and demographic tests using either microsatellite or mtDNA data.

	Microsatellite data		mtDNA—*COI*				mtDNA—*Cytb*			
	Bottleneck				Mismatch Distribution				Mismatch Distribution	
Population	TPM	SMM	Tajima’s *D*	Fu’s *Fs*	*SSD* (*P*_*SSD*_)	*Raggedness* (*P*_*r*_)	Tajima’s *D*	Fu’s *Fs*	*SSD* (*P*_*SSD*_)	*Raggedness* (*P*_*r*_)
Ti Point	0.741	0.945	-1.45	-10.8^c^	0.013 (0.225)	0.035 (0.298)	-1.31	-12.39^c^	0.007 (0.384)	0.053 (0.289)
Auckland	0.715	0.993	-2.16^b^	-25.26^c^	0.002 (0.537)	0.026 (0.417)	-1.90^a^	12.68^c^	0.006 (0.391)	0.047 (0.415)
Tauranga	0.689	0.995	-1.93^a^	-23.87^c^	0.003 (0.532)	0.015 (0.676)	-2.16^b^	-17.08^c^	0.010 (0.307)	0.053 (0.258)
Wellington	0.633	0.052	-1.68^a^	-11.66^c^	0.012 (0.212)	0.048 (0.215)	-1.38	-6.94^c^	0.003 (0.718)	0.029 (0.696)
Nelson	0.954	0.999	-2.09^b^	-24.73^c^	0.002 (0.761)	0.014 (0.847)	-2.17^b^	-26.33^c^	0.001 (0.672)	0.029 (0.588)
Total	-	-	-2.34^c^	-26.34^c^	0.003 (0.120)	0.032 (0.453)	-2.34^c^	-26.43^c^	0.003 (0.115)	0.032 (0.454)

Total values for Tajima’s *D*, Fu’s *Fs*, τ and the parameters of the mismatch distribution analysis were calculated pooling all available individuals into a single pool. TPM: Two-phase mutational model, SMM: stepwise mutational model, *SSD*: sum of squared deviations and statistical significance, *P*_*SSD*_: for the validity of the sudden expansion model, *τ*: time passed since population expansion, r*aggedness* and *P*_*r*_: Harpending’s raggedness index and its probability, respectively, for the null hypothesis test of goodness-of-fit.

*P*-values

^a^ <0.05

^b^ <0.01

^c^ <0.001

Isolation-by-migration models suggest that the northern and southern clusters split approx. 8.0 kya. The population sizes were estimated as 8.2 k in north, 11.4 k in south and 38.1 k in the ancestral population. The migration rate was slightly higher in the south (3.2 migrants per generation) than in the north (1.2 migrants per generation). Note, however, that the estimated 95% highest posterior density (HPD) intervals were wide for all parameters (e.g. the 95% HPD interval for the split time was 3.2–66.1 kya, and for the three population sizes (north, south and ancestral: 5.5–13.1 k, 7.7–17.0 k and 18.3–159.3 k, respectively).

#### Mitochondrial DNA analyses

**Fig 5:**
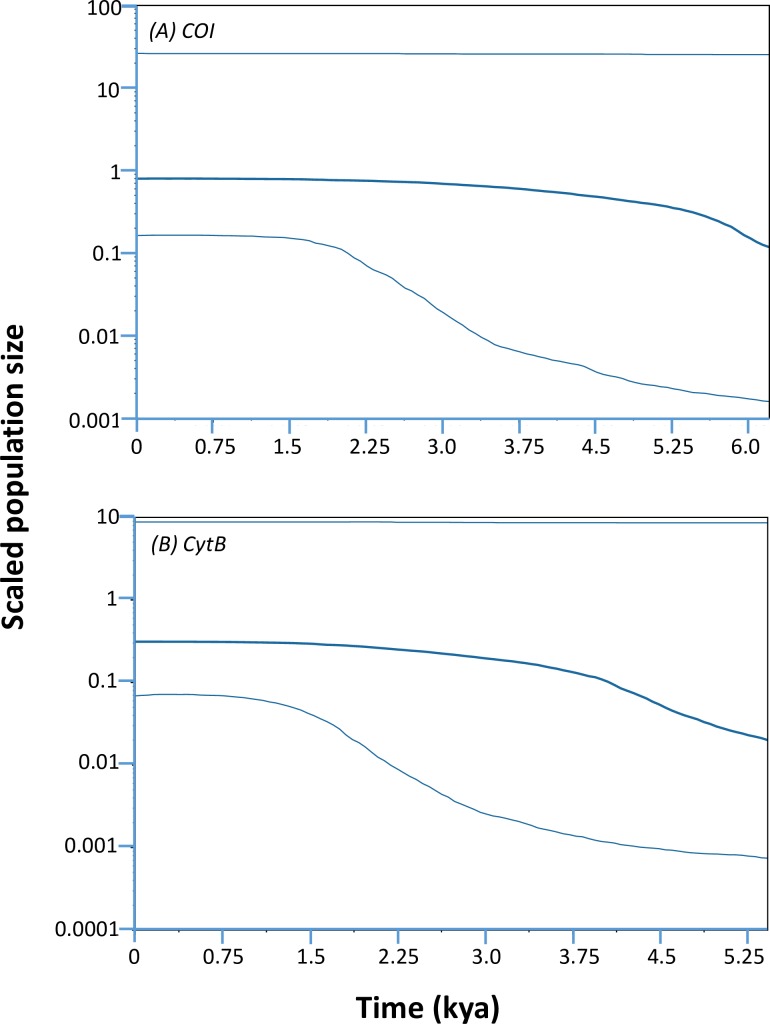
Bayesian skyline plots for mtDNA data. Plot for (A) *COI* and B) *Cytb* sequences. The middle line represents the median estimate of the effective population size, whereas the upper and lower lines represent the 95% highest posterior density.

Overall, neutrality tests of Tajima’s *D* and Fu’s *Fs* revealed significant negative values in pooled samples (Table 4) suggesting a recent population expansion or purifying selection. This was also suggested by the uni-modal mismatch distributions of pairwise base pair differences for *COI* and *CytB* haplotypes (Fig 4C and Figure G in S1 File, respectively), non-significant *SSD* and *raggedness* patterns (Table 4). Furthermore, the McDonald-Kreitman test found no evidence of positive selection: the ratio of nonsynonymous to synonymous substitutions within *P*. *maculata* (*Pn/Ps* = 5/136) and between species (*Dn/Ds* = 5/100) was statistically similar (neutrality index = 0.400, *P* = 0.1104). Reconstruction of the demographic history with Bayesian skyline plots showed signs of an expansion at around 5 kya for COI and CytB, but the 95% highest posterior density intervals are wide (Fig 5).

## Discussion

This study marks the first attempt to describe and account for patterns of genetic diversity in *P*. *maculata*. Overall, we observed marked signals of population structure; however, the population structure suggested by microsatellite versus mtDNA data differed. The nuclear data exhibit patterns of diversity indicative of a north-south disjunction. The northern samples formed one group and southern (WL and NL) samples formed another (with few examples of migration). However, this disjunction was not supported by *F*_*ST*_ analysis of mtDNA data, which indicated divergence among all populations.

Discordance between results obtained from nuclear versus mitochondrial markers is not uncommon, with explanations ranging from variation in lineage sorting to differences in rates of nuclear versus mitochondrial evolution. However, data from microsatellite markers that come from multiple unlinked nuclear loci are expected to provide a more accurate representation of population structure that mtDNA that are linked at a single locus [76]. Taken together, we interpret our data as indicative of a single founding population that subsequently became fragmented following geographical and oceanographical changes that led to the present north-south divide in NZ waters.

According to this scenario, *P*. *maculata* inhabited NZ waters before the end of the last glacial maximum (LGM) when sea levels were low and NZ was a single land mass [77, 78]. At some later time, most likely following the LGM (~22,000 years ago), large benthic habitats became available [79, 80], and this may have facilitated population expansion possibly from north to south and fragmentation aided by warming temperatures and rising sea levels [81].

In support of this model is the haplotype network based on mtDNA data showing star-like structure and high haplotype diversity, indicative of a population expansion arising from a small initial population [75]. Additional support comes from the unimodal mismatch distribution pattern of pairwise base pair differences for *COI* and *CytB* haplotypes (Fig 4C and Figure G in S1 File, respectively), non-significant *SSD* and *raggedness* patterns (Table 4), significant negative values for Tajima’s *D* and Fu’s *Fs*, and Bayesian Skyline plots (Fig 5). The approximate date of population expansion was estimated to be 5 kya. This estimate assumes a 2% divergence rate for the COI gene, which while an estimate, nonetheless suggests a recent expansion that could reasonably have followed the cyclic climatic oscillations defined by the late Pleistocene era (~110–15 kya) [82]. Glaciation has been suggested as the possible cause of demographic changes in other NZ marine organisms, including triplefin species [83], whelk species *Cominella virgata* and C. *maculosa* [84] and red alga *Bostrychia intricata* [85].

This scenario is also supported by the analysis of more rapidly evolving microsatellites, which are useful for uncovering recent barriers to gene flow [86–88]. As sea levels rose at the end of the last glacial cycle (~13,000 years ago; [~13,000 years ago; 78]), geographical factors and associated oceanography established barriers to gene flow for marine organisms. In particular, confluence of the East Cape current with the Wairapapa Eddy off the east coast of the North Island (between 37–39°S) created an oceanographic barrier [89]. A barrier was also formed by waters separating North and South Islands (the Cook Strait) [89, 90]. The split time (ca. 8 kya) estimated for the north and south *P*. *maculata* populations suggests that the genetic differentiation between the populations might have happened recently following creation of the present coastline after the last glacial maximum [77, 89]. The north-south disjunction identified for *P*. *maculata* can be explained by oceanographic barriers specific to NZ, but also with an isolation-by-distance model. The geographical gap between the sampling locations made it difficult to draw firm conclusions as to the origin of the disjunction.

A north-south genetic differentiation has been observed in other marine organisms from NZ (reviewed in [91, 92]). Confluence of the East Cape current with the Wairarapa Eddy is thought to be responsible for population differentiation in organisms such as the amphipods *Paracorophium excavatum* and *P*. *lucasi* [89] and the gastropod *Diloma subrostrata* [93]. The Cook Strait barrier is thought to have underpinned north-south differentiation in organisms such as the green shell mussel (*Perna canaliculus*) [94], blackfoot paua (*Haliotis iris*) [95] and the Ornate limpet (*Cellana ornate*) [96]. It may also explain the weak differentiation between WL and NL with connectivity between them being explained by the D’Urville Current that flows from the west into Cook Strait [97].

One additional factor that has likely promoted recent population subdivision in *P*. *maculata* is the distribution of invasive species [98] that constitute a food source for *P*. *maculata*. The Asian date mussel, *Arcuatula senhousia* has been established in the Auckland region since the 1970s, forming large transient beds in sub- and inter-tidal areas of the Hauraki Gulf, Manukau Harbour and Whangarei Harbour [99]. Expansion of *A*. *senhousia* beds in the Hauraki Gulf appears to have preceded increases in the density of *P*. *maculata* populations. Interestingly, subsequent decline of near-shore beds of *A*. *senhousia* post 2010 was followed by rapid decline in the density of *P*. *maculata* populations [100]. Further evidence that range expansion of *P*. *maculata* may have been facilitated by availability of prey species comes from off-shore mussel farms in Tasman Bay (Nelson, NZ), where culture of the green shell and blue mussels have created new habitats for *P*. *maculata*, with high-density populations being found beneath mussel farms [100]. Recently, *P*. *maculata* was identified in Argentinean waters with the species rapidly spreading along the Atlantic coast [6, 7]. The minor difference between mtDNA sequence in slugs from Argentina versus NZ raises the possibility that NZ maybe the source of the recently discovered population in Argentina.

Life history traits such as the nature of egg and larval stages are of understandable importance in shaping population structure of the species. Species with benthic eggs tend to have more structured populations than ones with pelagic eggs [101, 102] and an inverse relationship between pelagic larval duration (PLD) and genetic structure has been found [92, 103]: in a comparative analysis of NZ pelagic marine species, Ross et al. [92] showed a significant negative correlation between PLD and genetic differentiation. However, in the same meta-study, when NZ-wide sampling regimes were considered, NZ organisms with PLD durations similar to *P*. *maculata* (2–4 weeks) exhibit structural patterns of diverse types ranging from no structure, to a north-south disjunction, IBD and differentiation within and between sampling locations [92]. The three-week pelagic larval stage of *P*. *maculata* [3, 4, 91] likely confers a high dispersal capacity on the species. Migration analysis identified first generation migrants between the two clusters. However, the north-south disjunction still shows that dispersal is limited. Beyond barriers formed via ocean currents, density-dependent processes acting at regional scales may act to limit invasion by new types [104].

Our prediction that the previously recorded cline in TTX might be explained by genetic structure holds only for microsatellite markers. Had this also held for mtDNA markers a case may have been made that *P*. *maculata* is a complex of two cryptic species, but no such evidence exists, at least for the samples included into this study. Our phylogenetic analysis indicates that all *P*. *maculata* populations–including samples from Argentina waters [6]–are conspecific. Short branches with no or low bootstrap support is also indicative of lack of genetic differentiation among *P*. *maculata*. Similar lack of differentiation between toxic and non-toxic populations has been shown for *Taricha granulosa* newts from various localities in western North America [105, 106] and the red-spotted newt *Notophthalmus viridescens* [107].

Having called into question substantive genetic differences between north and south populations, differences in TTX levels are thus likely attributable to exogenous factors, such as differences in associated bacteria, exposure, or diet. Work to date is strongly suggestive of diet as the major source of TTX, with *P*. *maculata* accumulating TTX via feeding [11], while offspring from TTX positive individuals raised in a TTX-free environment become free of TTX [13]. Recent work studying cultured bacteria from *P*. *maculata* found no evidence for a bacterial origin of the toxin [16], but TTX has been reported in certain prey, including a Platyhelminthes *Stylochoplana* species that co-occurs with TTX-containing *P*. *maculata* [12].

## Supporting information

S1 File Supplementary methodology and results. (DOCX)

## References

[1] Willan R. New-Zealand side-gilled sea slugs (Opisthobranchia, Notaspidea, Pleurobranchidae). Malacologia. 1983;23(2):221–70.

[2] Bökenhans V, Fernández Alfaya JE, Bigatti G, Averbuj A. Diet of the invasive sea slug *Pleurobranchaea maculata* in Patagonian coastal waters. New Zeal J Zool. 2018:1–8.

[3] Wood SA, Taylor DI, McNabb P, Walker J, Adamson J, Cary SC. Tetrodotoxin concentrations in *Pleurobranchaea maculata*: temporal, spatial and individual variability from New Zealand populations. Mar Drugs. 2012;10(1):163–76. 10.3390/md10010163 22363228 PMC3280535

[4] Gibson GD. Larval development and metamorphosis in *Pleurobranchaea maculata*, with a review of development in the Notaspidea (Opisthobranchia). Bio Bull. 2003;205(2):121–32. 14583510 10.2307/1543233

[5] McNabb P, Selwood AI, Munday R, Wood SA, Taylor DI, Mackenzie LA, et al Detection of tetrodotoxin from the grey side-gilled sea slug—*Pleurobranchaea maculata*, and associated dog neurotoxicosis on beaches adjacent to the Hauraki Gulf, Auckland, New Zealand. Toxicon. 2010;56(3):466–73. 10.1016/j.toxicon.2010.04.017 20466016

[6] Farias N, Wood S, Obenat S, Schwindt E. Genetic barcoding confirms the presence of the neurotoxic sea slug *Pleurobranchaea maculata* in southwestern Atlantic coast. New Zeal J Zool. 2016;43(3):292–8.

[7] Farias NE, Obenat S, Goya AB. Outbreak of a neurotoxic side-gilled sea slug (*Pleurobranchaea* sp.) in Argentinian coasts. New Zeal J Zool. 2015;42(1):51–6.

[8] Noguchi T, Arakawa O. Tetrodotoxin—Distribution and accumulation in aquatic organisms, and cases of human intoxication. Mar Drugs. 2008;6(2):220–42. 10.3390/md20080011 18728726 PMC2525488

[9] Magarlamov TY, Melnikova DI, Chernyshev AV. Tetrodotoxin-producing bacteria: detection, distribution and migration of the toxin in aquatic systems. Toxins. 2017;9(5). 10.3390/toxins9050166 PMC5450714 28513564

[10] Matsumura K. Reexamination of tetrodotoxin production by bacteria. Appl Environ Microb. 1995;61(9):3468–70. 10.1128/aem.61.9.3468-3470.1995 PMC167625 7574655

[11] Khor S, Wood SA, Salvitti L, Taylor DI, Adamson J, McNabb P, et al Investigating diet as the source of tetrodotoxin in *Pleurobranchaea maculata*. Mar Drugs. 2014;12(1):1–16. 10.3390/md12010001 PMC3917257 24368566

[12] Salvitti L, Wood SA, Taylor DI, McNabb P, Cary SC. First identification of tetrodotoxin (TTX) in the flatworm *Stylochoplana* sp.; a source of TTX for the sea slug *Pleurobranchaea maculata*. Toxicon. 2015;95:23–9. 10.1016/j.toxicon.2014.12.006 25557071

[13] Wood SA, Casas M, Taylor DI, McNabb P, Salvitti L, Ogilvie S, et al Depuration of tetrodotoxin and changes in bacterial communities in *Pleurobranchea maculata* adults and egg masses maintained in captivity. J Chem Ecol. 2012;38(11):1342–50. 10.1007/s10886-012-0212-9 23151964

[14] Salvitti L, Wood SA, Fairweather R, Culliford D, McNabb P, Cary SC. In situ accumulation of tetrodotoxin in non-toxic *Pleurobranchaea maculata* (Opisthobranchia). Aquat Sci. 2017;79(2):335–44.

[15] Chau R, Kalaitzis JA, Wood SA, Neilan BA. Diversity and biosynthetic potential of culturable microbes associated with toxic marine animals. Mar Drugs. 2013;11(8):2695–712. 10.3390/md11082695 23917066 PMC3766859

[16] Salvitti LR, Wood SA, McNabb P, Cary SC. No evidence for a culturable bacterial tetrodotoxin producer in *Pleurobranchaea maculata* (Gastropoda: Pleurobranchidae) and *Stylochoplana* sp. (Platyhelminthes: Polycladida). Toxins (Basel). 2015;7(2):255–73. 25635464 10.3390/toxins7020255 PMC4344623

[17] Yıldırım Y, Patel S, Millar CD, Rainey PB. Microsatellite development for a tetrodotoxin-containing sea slug (*Pleurobranchaea maculata*). Biochem Syst Ecol. 2014;55:342–5.

[18] Van Oosterhout C, Hutchinson WF, Wills DP, Shipley P. MICRO‐CHECKER: software for identifying and correcting genotyping errors in microsatellite data. Mol Ecol Notes. 2004;4(3):535–8.

[19] Weir BS, Cockerham CC. Estimating F-statistics for the analysis of population structure. Evolution. 1984:1358–70. 10.1111/j.1558-5646.1984.tb05657.x 28563791

[20] Meirmans PG, Van Tienderen PH. GENOTYPE and GENODIVE: two programs for the analysis of genetic diversity of asexual organisms. Mol Ecol Notes. 2004;4(4):792–4.

[21] Benjamini Y, Hochberg Y. Controlling the false discovery rate—a practical and powerful approach to multiple testing. J R Stat Soc Series B Methodol. 1995;57(1):289–300.

[22] Goudet J. FSTAT (Version 1.2): A computer program to calculate F-statistics. J Hered. 1995;86(6):485–6.

[23] Wright S. Isolation by distance under diverse systems of mating. Genetics. 1946;31(1):39–59. 21009706 10.1093/genetics/31.1.39 PMC1209315

[24] Wright S. The genetical structure of populations. Ann Hum Genet. 1949;15(1):323–54. 10.1111/j.1469-1809.1949.tb02451.x 24540312

[25] Wright S. The interpretation of population structure by F‐statistics with special regard to systems of mating. Evolution. 1965;19(3):395–420.

[26] Peakall R, Smouse PE. GENALEX 6: genetic analysis in Excel. Population genetic software for teaching and research. Mol Ecol Notes. 2006;6(1):288–95. 10.1093/bioinformatics/bts460 PMC3463245 22820204

[27] Szpiech ZA, Jakobsson M, Rosenberg NA. ADZE: a rarefaction approach for counting alleles private to combinations of populations. Bioinformatics. 2008;24(21):2498–504. 10.1093/bioinformatics/btn478 18779233 PMC2732282

[28] Marx A, Backes C, Meese E, Lenhof H-P, Keller A. EDISON-WMW: Exact Dynamic Programing Solution of the Wilcoxon–Mann–Whitney Test. Genomics Proteomics Bioinformatics. 2016;14(1):55–61. 10.1016/j.gpb.2015.11.004 26829645 PMC4792850

[29] Tajima F. Evolutionary relationship of DNA sequences in finite populations. Genetics. 1983;105(2):437–60. 6628982 10.1093/genetics/105.2.437 PMC1202167

[30] Nei M. Molecular Evolutionary Genetics. New York: Columbia University Press; 1987.

[31] Librado P, Rozas J. DnaSP v5: a software for comprehensive analysis of DNA polymorphism data. Bioinformatics. 2009;25(11):1451–2. 10.1093/bioinformatics/btp187 19346325

[32] Raymond M, Rousset F. An exact test for population differentiation. Evolution. 1995;49(6):1280–3. 10.1111/j.1558-5646.1995.tb04456.x 28568523

[33] Jost L. GST and its relatives do not measure differentiation. Mol Ecol. 2008;17(18):4015–26. 19238703 10.1111/j.1365-294x.2008.03887.x

[34] Ryman N, Palm S. POWSIM: a computer program for assessing statistical power when testing for genetic differentiation. Mol Ecol Notes. 2006;6(3):600–2.

[35] Pritchard JK, Stephens M, Donnelly P. Inference of population structure using multilocus genotype data. Genetics. 2000;155(2):945–59. 10835412 10.1093/genetics/155.2.945 PMC1461096

[36] Evanno G, Regnaut S, Goudet J. Detecting the number of clusters of individuals using the software STRUCTURE: a simulation study. Mol Ecol. 2005;14(8):2611–20. 10.1111/j.1365-294X.2005.02553.x 15969739

[37] Earl DA. STRUCTURE HARVESTER: a website and program for visualizing STRUCTURE output and implementing the Evanno method. Conserv Genet Resour. 2012;4(2):359–61.

[38] Jakobsson M, Rosenberg NA. CLUMPP: a cluster matching and permutation program for dealing with label switching and multimodality in analysis of population structure. Bioinformatics. 2007;23(14):1801–6. 10.1093/bioinformatics/btm233 17485429

[39] Rosenberg NA. DISTRUCT: a program for the graphical display of population structure. Mol Ecol Notes. 2004;4(1):137–8.

[40] Bandelt HJ, Forster P, Röhl A. Median-joining networks for inferring intraspecific phylogenies. Mol Biol Evol. 1999;16(1):37–48. 10.1093/oxfordjournals.molbev.a026036 10331250

[41] Xia X. DAMBE5: a comprehensive software package for data analysis in molecular biology and evolution. Mol Biol Evol. 2013;30(7):1720–8. 10.1093/molbev/mst064 23564938 PMC3684854

[42] Xia X, Xie Z, Salemi M, Chen L, Wang Y. An index of substitution saturation and its application. Mol Phylogenet Evol. 2003;26(1):1–7. 12470932 10.1016/s1055-7903(02)00326-3

[43] Posada D. jModelTest: Phylogenetic Model Averaging. Mol Biol Evol. 2008;25(7):1253–6. 10.1093/molbev/msn083 18397919

[44] Nei M. Estimation of average heterozygosity and genetic distance from a small number of individuals. Genetics. 1978;89(3):583–90. 17248844 10.1093/genetics/89.3.583 PMC1213855

[45] Excoffier L, Smouse PE, Quattro JM. Analysis of molecular variance inferred from metric distances among DNA haplotypes: application to human mitochondrial DNA restriction data. Genetics. 1992;131(2):479–91. 1644282 10.1093/genetics/131.2.479 PMC1205020

[46] Excoffier L, Laval G, Schneider S. Arlequin (version 3.0): An integrated software package for population genetics data analysis. Evol Bioinform. 2005;1:47–50. PMC2658868 19325852

[47] Tamura K, Nei M. Estimation of the number of nucleotide substitutions in the control region of mitochondrial DNA in humans and chimpanzees. Mol Biol Evol. 1993;10(3):512–26. 10.1093/oxfordjournals.molbev.a040023 8336541

[48] Schloss PD, Westcott SL, Ryabin T, Hall JR, Hartmann M, Hollister EB, et al Introducing mothur: open-source, platform-independent, community-supported software for describing and comparing microbial communities. Appl Environ Microb. 2009;75(23):7537–41. 10.1128/AEM.01541-09 PMC2786419 19801464

[49] Clarke K, Gorley R. PRIMER v6: User Manual/Tutorial: PRIMER-E Ltd: Plymouth, UK; 2006.

[50] Anderson MJ, Gorley RN, Clarke KR. PERMANOVA+ for PRIMER: Guide to Software and Statistical Methods: PRIMER-E: Plymouth, UK; 2008.

[51] Kruskal JB. Nonmetric multidimensional scaling: a numerical method. Psychometrika. 1964;29(2):115–29.

[52] Anderson MJ. A new method for non‐parametric multivariate analysis of variance. Austral Ecol. 2001;26(1):32–46.

[53] McArdle BH, Anderson MJ. Fitting multivariate models to community data: a comment on distance‐based redundancy analysis. Ecology. 2001;82(1):290–7.

[54] Anderson MJ, Willis TJ. Canonical analysis of principal coordinates: a useful method of constrained ordination for ecology. Ecology. 2003;84(2):511–25.

[55] Anderson MJ. Distance‐based tests for homogeneity of multivariate dispersions. Biometrics. 2006;62(1):245–53. 10.1111/j.1541-0420.2005.00440.x 16542252

[56] Kumar S, Stecher G, Tamura K. MEGA7: Molecular Evolutionary Genetics Analysis Version 7.0 for Bigger Datasets. Mol Biol Evol. 2016;33(7):1870–4. 10.1093/molbev/msw054 27004904 PMC8210823

[57] Piry S, Alapetite A, Cornuet J-M, Paetkau D, Baudouin L, Estoup A. GENECLASS2: a software for genetic assignment and first-generation migrant detection. J Hered. 2004;95(6):536–9. 10.1093/jhered/esh074 15475402

[58] Rannala B, Mountain JL. Detecting immigration by using multilocus genotypes. Proc Natl Acad Sci U S A. 1997;94(17):9197–201. 9256459 10.1073/pnas.94.17.9197 PMC23111

[59] Paetkau D, Slade R, Burden M, Estoup A. Genetic assignment methods for the direct, real‐time estimation of migration rate: a simulation‐based exploration of accuracy and power. Mol Ecol. 2004;13(1):55–65. 14653788 10.1046/j.1365-294x.2004.02008.x

[60] Piry S, Luikart G, Cornuet JM. BOTTLENECK: a computer program for detecting recent reductions in the effective size using allele frequency data. J Hered. 1999;90(4):502–3.

[61] Luikart G, Cornuet JM. Empirical evaluation of a test for identifying recently bottlenecked populations from allele frequency data. Conserv Biol. 1998;12(1):228–37.

[62] Hey J, Nielsen R. Integration within the Felsenstein equation for improved Markov chain Monte Carlo methods in population genetics. Proc Natl Acad Sci U S A. 2007;104(8):2785–90. 10.1073/pnas.0611164104 17301231 PMC1815259

[63] Hey J. Isolation with migration models for more than two populations. Mol Biol Evol. 2010;27(4):905–20. 10.1093/molbev/msp296 19955477 PMC2877539

[64] Jarne P, Lagoda PJL. Microsatellites, from molecules to populations and back. Trends Ecol Evol. 1996;11(10):424–9. 21237902 10.1016/0169-5347(96)10049-5

[65] Muñoz MM, Crawford NG, Mcgreevy TJ, Messana NJ, Tarvin RDs, Revell LJ, et al Divergence in coloration and ecological speciation in the *Anolis marmoratus* species complex. Mol Ecol. 2013;22(10):2668–82. 10.1111/mec.12295 23611648

[66] Tajima F. Statistical method for testing the neutral mutation hypothesis by DNA polymorphism. Genetics. 1989;123(3):585–95. 2513255 10.1093/genetics/123.3.585 PMC1203831

[67] Fu Y-X. Statistical tests of neutrality of mutations against population growth, hitchhiking and background selection. Genetics. 1997;147(2):915–25. 9335623 10.1093/genetics/147.2.915 PMC1208208

[68] Schneider S, Excoffier L. Estimation of past demographic parameters from the distribution of pairwise differences when the mutation rates vary among sites: application to human mitochondrial DNA. Genetics. 1999;152(3):1079–89. 10388826 10.1093/genetics/152.3.1079 PMC1460660

[69] Harpending H. Signature of ancient population growth in a low-resolution mitochondrial DNA mismatch distribution. Hum Biol. 1994:591–600. 8088750

[70] McDonald JH, Kreitman M. Adaptive protein evolution at the Adh locus in *Drosophila*. Nature. 1991;351(6328):652 10.1038/351652a0 1904993

[71] Ho SYW, Shapiro B. Skyline-plot methods for estimating demographic history from nucleotide sequences. Mol Ecol Resourc. 2011;11(3):423–34. 10.1111/j.1755-0998.2011.02988.x 21481200

[72] Bouckaert R, Heled J, Kuhnert D, Vaughan T, Wu CH, Xie D, et al BEAST 2: A software platform for Bayesian evolutionary analysis. Plos Comput Biol. 2014;10(4). 10.1371/journal.pcbi.1003537 PMC3985171 24722319

[73] Crandall ED, Sbrocco EJ, DeBoer TS, Barber PH, Carpenter KE. Expansion dating: calibrating molecular clocks in marine species from expansions onto the Sunda Shelf following the Last Glacial Maximum. Mol Biol Evol. 2011;29(2):707–19. 10.1093/molbev/msr227 21926069

[74] Rambaut A, Drummond AJ, Xie D, Baele G, Suchard MA. Posterior summarisation in Bayesian phylogenetics using Tracer 1.7. Syst Biol. 2018;10. 10.1093/sysbio/syy032 PMC6101584 29718447

[75] Slatkin M, Hudson RR. Pairwise comparisons of mitochondrial DNA sequences in stable and exponentially growing populations. Genetics. 1991;129(2):555–62. 1743491 10.1093/genetics/129.2.555 PMC1204643

[76] Edwards S, Bensch S. Looking forwards or looking backwards in avian phylogeography? A comment on Zink and Barrowclough 2008. Mol Ecol. 2009;18(14):2930–3. 10.1111/j.1365-294X.2009.04270.x 19552688

[77] Lewis KB, Carter L, Davey FJ. The opening of Cook Strait: interglacial tidal scour and aligning basins at a subduction to transform plate edge. Mar Geol. 1994;116(3–4):293–312.

[78] Trewick S, Bland K. Fire and slice: palaeogeography for biogeography at New Zealand's North Island/South Island juncture. J Roy Soc New Zeal. 2012;42(3):153–83.

[79] Allcock AL, Strugnell JM. Southern Ocean diversity: new paradigms from molecular ecology. Trends Ecol Evol. 2012;27(9):520–8. 10.1016/j.tree.2012.05.009 22727016

[80] Norris RD, Hull PM. The temporal dimension of marine speciation. Evol Ecol. 2012;26(2):393–415.

[81] Weaver PP, Carter L, Neil HL. Response of surface water masses and circulation to late Quaternary climate change east of New Zealand. Paleoceanography. 1998;13(1):70–83.

[82] Gradstein FM, Ogg JG, Smith AG, Bleeker W, Lourens LJ. A new geologic time scale, with special reference to Precambrian and Neogene. Episodes. 2004;27(2):83–100.

[83] Hickey AJ, Lavery SD, Hannan DA, Baker CS, Clements KD. New Zealand triplefin fishes (family Tripterygiidae): contrasting population structure and mtDNA diversity within a marine species flock. Mol Ecol. 2009;18(4):680–96. 10.1111/j.1365-294X.2008.04052.x 19215584

[84] Fleming AM, Dohner MM, Phillips NE, Ritchie PA. Genetic connectivity among populations of two congeneric direct-developing whelks varies across spatial scales. New Zeal J Mar Fresh. 2018;52(1):100–17.

[85] Muangmai N, Fraser CI, Zuccarello GC. Contrasting patterns of population structure and demographic history in cryptic species of *Bostrychia intricata* (Rhodomelaceae, Rhodophyta) from New Zealand. J Phycol. 2015;51(3):574–85. 10.1111/jpy.12305 26986671

[86] Barr KR, Lindsay DL, Athrey G, Lance RF, Hayden TJ, Tweddale SA, et al Population structure in an endangered songbird: maintenance of genetic differentiation despite high vagility and significant population recovery. Mol Ecol. 2008;17(16):3628–39. 10.1111/j.1365-294X.2008.03868.x 18643883

[87] Edwards S, Bensch S. Looking forwards or looking backwards in avian phylogeography? A comment on. Mol Ecol. 2009;18(14):2930–3. 10.1111/j.1365-294X.2009.04270.x 19552688

[88] Zink RM, Groth JG, Vázquez-Miranda H, Barrowclough GF. Phylogeography of the California Gnatcatcher (*Polioptila californica*) using multilocus DNA sequences and ecological niche modeling: Implications for conservation. The Auk. 2013;130(3):449–58.

[89] Stevens MI, Hogg ID. Population genetic structure of New Zealand's endemic corophiid amphipods: evidence for allopatric speciation. Biol J Linnean Soc. 2004;81(1):119–33.

[90] Keeney DB, Szymaniak AD, Poulin R. Complex genetic patterns and a phylogeographic disjunction among New Zealand mud snails *Zeacumantus subcarinatus* and *Z*. *lutulentus*. Mar Biol. 2013;160(6):1477–88.

[91] Gardner J, Bell J, Constable H, Hannan D, Ritchie P, Zuccarello G. Multi-species coastal marine connectivity: a literature review with recommendations for further research. New Zeal Aquat Environ Biodiversity Rep. 2010;58:1–47.

[92] Ross PM, Hogg ID, Pilditch CA, Lundquist CJ. Phylogeography of New Zealand's coastal benthos. New Zeal J Mar Fresh. 2009;43(5):1009–27.

[93] Donald KM, Kennedy M, Spencer HG. Cladogenesis as the result of long-distance rafting events in South Pacific topshells (Gastropoda, Trochidae). Evolution. 2005;59(8):1701–11. 16329241

[94] Wei KJ, Wood AR, Gardner JPA. Population genetic variation in the New Zealand greenshell mussel: locus-dependent conflicting signals of weak structure and high gene flow balanced against pronounced structure and high self-recruitment. Mar Biol. 2013;160(4):931–49.

[95] Waters JM, King TM, O'Loughlin PM, Spencer HG. Phylogeographical disjunction in abundant high-dispersal littoral gastropods. Mol Ecol. 2005;14(9):2789–802. 10.1111/j.1365-294X.2005.02635.x 16029478

[96] Goldstien SJ, Schiel DR, Gemmell NJ. Comparative phylogeography of coastal limpets across a marine disjunction in New Zealand. Mol Ecol. 2006;15(11):3259–68. 10.1111/j.1365-294X.2006.02977.x 16968269

[97] Heath R. What drives the mean circulation on the New Zealand west coast continental shelf? New Zeal J Mar Fresh. 1982;16(2):215–26.

[98] Rodriguez LF. Can invasive species facilitate native species? Evidence of how, when, and why these impacts occur. Biol Invasions. 2006;8(4):927–39.

[99] Crooks JA. Characterizing ecosystem‐level consequences of biological invasions: the role of ecosystem engineers. Oikos. 2002;97(2):153–66.

[100] Taylor DI, Wood SA, McNabb P, Ogilvie S, Cornelisen C, Walker J, et al Facilitation effects of invasive and farmed bivalves on native populations of the sea slug *Pleurobranchaea maculata*. Mar Ecol Prog Ser. 2015;537:39–48.

[101] Riginos C, Douglas KE, Jin Y, Shanahan DF, Treml EA. Effects of geography and life history traits on genetic differentiation in benthic marine fishes. Ecography. 2011;34(4):566–75.

[102] Riginos C, Buckley YM, Blomberg SP, Treml EA. Dispersal capacity predicts both population genetic structure and species richness in reef fishes. Am Nat. 2014;184(1):52–64. 10.1086/676505 24921600

[103] Selkoe KA, Toonen RJ. Marine connectivity: a new look at pelagic larval duration and genetic metrics of dispersal. Mar Ecol Prog Ser. 2011;436:291–305.

[104] Waters JM, Fraser CI, Hewitt GM. Founder takes all: density-dependent processes structure biodiversity. Trends Ecol Evol. 2013;28(2):78–85. 10.1016/j.tree.2012.08.024 23000431

[105] Hanifin CT, Brodie ED Jr, Brodie III ED. Phenotypic mismatches reveal escape from arms-race coevolution. PLoS Biol. 2008;6(3):e60 10.1371/journal.pbio.0060060 18336073 PMC2265764

[106] Ridenhour BJ, Brodie ED Jr, Brodie III ED. Patterns of genetic differentiation in *Thamnophis* and *Taricha* from the Pacific Northwest. J Biogeogr. 2007;34(4):724–35.

[107] Yotsu-Yamashita M, Gilhen J, Russell RW, Krysko KL, Melaun C, Kurz A, et al Variability of tetrodotoxin and of its analogues in the red-spotted newt, *Notophthalmus viridescens* (Amphibia: Urodela: Salamandridae). Toxicon. 2012;59(2):257–64. 10.1016/j.toxicon.2011.12.004 22197660

