# Supplementary material for: Genetic structure of the grey side-gilled sea slug (Pleurobranchaea maculata) in coastal waters of New Zealand
Source: PLoS One. 2018 Aug 16;13(8):e0202197. doi: 10.1371/journal.pone.0202197 (PMC6095540; doi:10.1371/journal.pone.0202197)
Supplement: S1 File — (DOCX) [file pone.0202197.s001.docx]

**Genetic structure of the grey side-gilled sea slug (*Pleurobranchaea maculata*) in coastal waters of New Zealand** – Supplementary file

Yeşerin Yıldırım^1,#a*^, Marti J. Anderson^1,2^, Bengt Hansson^3^, Selina Patel^4^, Craig D. Millar^4^, and Paul B. Rainey^1,5,6^

^1^New Zealand Institute for Advanced Study, Massey University, Auckland, New Zealand; ^2^Institute of Natural and Mathematical Sciences, Massey University, Auckland, New Zealand; ^3^Department of Biology, Lund University, Lund, Sweden; ^4^School of Biological Sciences, University of Auckland, Auckland, New Zealand; ^5^Department of Microbial Population Biology, Max Planck Institute for Evolutionary Biology, Plön, Germany; ^6^Ecole Supérieure de Physique et de Chimie Industrielles de la Ville de Paris (ESPCI ParisTech), CNRS UMR 8231, PSL Research University, Paris, France; Current Address: ^#a^Center for Ecology and Evolution in Microbial Systems (EEMiS), Department of Biology and Environmental Science, Linnaeus University, Kalmar, Sweden.

# Supplementary methodology and results

## Methodology

**Table A. Sampling locations and abbreviations for the New Zealand *Pleurobranchaea maculata* populations.**

| **# of samples (mtDNA/microsats)*** | **Location** | **Longitude** | **Latitude** | **Sampling date** |
| --- | --- | --- | --- | --- |
| 5 /1 | Urquharts Bay, Whangarei (WH) | -35.842675 | 174.527764 | Aug 2010 |
| 20 /20 | Whangateau Harbour, Ti Point (TP) | -36.3175 | 174.784444 | Aug 2012 |
| 3 /1 | Whangapoua Harbour, Coromandel (CR) | -36.741281 | 175.627905 | Sep 2012 |
| 19 /19 | Tamaki Strait, Auckland (AKL) | -36.781333 | 174.824473 | Jul 2011/Oct 2012 |
| 1 /1 | Tamaki Strait, Auckland (AKL) | -36.81104 | 174.800399 | Winter 2009 |
| 7 /7 | Waitemata Harbour, Auckland (AKL) | -36.82888 | 174.76458 | Oct 2012 |
| 4 /4 | Waitemata Harbour, Auckland (AKL) | -36.853077 | 174.712449 | Oct 2012 |
| 20 /20 | Tauranga Harbour, Tauranga (TR) | -37.634465 | 176.176455 | Jun 2011/Jun 2012 |
| 6 /6 | Tauranga Harbour, Tauranga (TR) | -37.677623 | 176.1766 | Jun 2013 |
| 7 /7 | Tauranga Harbour, Tauranga (TR) | -37.643948 | 176.148448 | Oct 2013 |
| 18 /18 | Lambton Harbour, Wellington (WL) | -41.288433 | 174.779983 | Oct 2012 |
| 45 /45 | Tasman Bay, Nelson (NL) | -41.058056 | 173.091111 | Nov 2010-Aug 2013 |
| 1 /1 | Picton Harbour, Picton (PC) | -41.272714 | 174.013433 | Dec 2012 |
| 1 /0 | Kaikoura (KK) | -42.465567 | 173.781517 | Nov 2011 |

* Due to the DNA degradation problem, some samples microsatellite markers could not be amplified for all individuals. The number of individuals genotyped for each marker type is indicated.

### Amplification of microsatellite markers

The 5’ ends of the forward primers of the microsatellite markers were tagged with a 5’-fluorescent dye (6-FAM, VIC, NED and PET) except *Pm19* that was tagged with a universal M13(-21) sequence (18bp) [1]. For the primer tagged with the M13(-21) sequence, a third primer with the M13(-21)-sequence and a 5’-fluorescent dye (6-FAM) were used for amplification. The primers for the former loci were pooled into three multiplex sets; polymerase chain reaction (PCR) was used to amplify the DNA for each individual. For directly labelled primer pairs, PCRs were carried with 1X Qiagen Multiplex PCR Master Mix (Qiagen, Heiden, Germany), 1X Qiagen Q-buffer, RNase-free water, primer mix (*Pm 09* and *Pm10* at 0.025 μM, *Pm20* at 0.05 μM, the rest of the primers at 0.1 μM), and 10–25 ng genomic DNA. For *Pm19*, the PCR was carried out with a 1X PCR buffer (Invitrogen Taq DNA Polymerase, recombinant, Invitrogen, USA), 2 mM MgCl2, 0.4 μM M13(-21) primer and reverse primer, 0.1 μM forward primer, 0.2 mM dNTP, 0.02 U/mL Invitrogen Taq polymerase and 10–25 ng genomic DNA. Either standard or touch-down PCR regimes were applied (Supplementary Table 1). Standard conditions were as follows: 94°C for 3 minutes for *Pm19* or 95°C for 15 minutes for the directly labelled primers, then 30 cycles at 94°C (30 s)/Tm (30 s)/72°C (60 s). Touch-down conditions were as follows: 94°C (3 min), then 20 cycles at 94°C (30 s)/63°C (decreasing by 0.5°C/cycle, 30 s)/72°C (30–60 s) followed by 20 cycles at 94°C (30 s)/53°C (30 s)/72°C (30–60 s). The regime was followed by 8 cycles at 94°C (30 s)/53°C (30 s)/72°C (20–60 s) for *Pm19*. A final extension at 72°C was applied for all reactions for 30 or 10 minutes, depending on using Multiplex PCR buffer or not, respectively. The PCR regime, annealing temperature and elongation time were estimated according to the primer pair properties, and these thermocycling conditions are shown in Supplementary Table 1. The multiplexed amplicons were electrophoresed on a commercial ABI 3730 Genetic Analyzer along with a size standard GeneScan™-500 LIZ® (Applied Biosystems). The resulting genotyping data were analysed with a Microsatellite Plug-in in Geneious Pro 5.5.6 (Biomatters, New Zealand). The genotyping data were imported into GenAlex 6.5 [2], which is an Excel Add-In to convert the data into the necessary format for further analysis. Alternatively, PGDSPIDER V2.0.7.2 [3] was used for the data conversions.

**Table B. PCR annealing temperature and elongation time for microsatellite markers.**

| Locus ID | Annealing temperature (˚C) | Elongation time (sec) |
| --- | --- | --- |
| *Pm01*, *Pm08*, *Pm09*, *Pm10*, †*Pm19* | 57 | 60 |
| *Pm02*, *Pm07*, *Pm11,* *Pm13*, *Pm17* | 63 to 53* | 60 |
| *Pm20*, *Pm23* | 63 to 53* | 30 |

* Touchdown regime. †Forward primer was tagged with a universal M13(-21) sequence; a third primer with the M13(-21)-sequence and tagged with a 5’-fluorescent dye (6-FAM) were used for amplification. Otherwise, forward primers were tagged with a fluorescent dye.

### Amplification of mtDNA markers

Mitochondrial DNA sequences for the cytochrome B (*CytB*) and co-oxidase 1 (*COI*) genes of three Nudipleuroubranch species, *Notodoris gardineri*, *Chromodis* *magnifica* and *Berthellina* sp. TLT-2006 [4], and partial COI sequences of *P. maculata* [5] were obtained from Genbank. The sequences were aligned using the Geneious multiple alignment tool, and several primer pairs were designed using the most conserved regions to cover as much of the genes as possible with Primer in Geneious 5.6.5. The list of the mtDNA primers used during this study can be found in Supplementary Table 2. In addition, universal *COI* [6], 16S *rRNA* [7] and universal *cytB* primers from Merritt *et al*. [8] which were modified in this study based on Nudipleuroubranch alignment were used to amplify fragments of the genes. Universal COI primer pair did not work. Therefore, only universal reverse *COI* primer from Folmer *et al* [6] could be used. To check if these genes are polymorphic for *P. maculata*, the gene fragments were amplified first in seven individuals sampled from various locations: two from AKL and one from each of the following locations: TP, TR, WL, NL and PC. The sequence data were aligned to identify the polymorphic regions for *P. maculata*, and species-specific primer pairs were designed to amplify the polymorphic regions of the *COI* and *CytB* genes. *16S rRNa* gene was not used for further amplifications due to the lack of polymorphism among the seven individuals. Two primer pairs were designed to amplify the 5’ and the 3’ halves of the variable regions for both genes, separately. These primer pairs were used to genotype the remaining 149 individuals. The PCR for each pair of primers was carried out in a total volume of 50 µL, including 1X Invitrogen™ *Taq* DNA Polymerase buffer, 2 mM MgCl_2_, 0.2 mM dNTPs, 4 mM tetramethylammonium chloride, 1X bovine serum albumin, 0.4 µM from each of the forward and reverse primers, 0.02 U Invitrogen™ Taq DNA Polymerase, and 30–90 ng genomic DNA. Either standard or touchdown PCR regimes were applied. Standard conditions were as follows: 94°C (3 min), then 30 cycles at 94°C (30 s)/Tm (30 s)/72°C (80 s). Touch-down conditions were as follows: 94°C (3 min), then 15 cycles at 94°C (30 s)/ 61.5°C (decreasing by 0.5°C/cycle, 30s)/72°C (80 s) followed by 20 cycles at 94°C (30s)/53°C (30s)/72°C (80 s). A final extension at 72°C was applied for all reactions for 6 minutes (Please see Supplementary Table 3 for thermocyling conditions). The samples were sequenced from both 5’ and 3’ ends using Sanger Sequencing techniques by Macrogen Inc. (Korea). Geneious 6.1.6 was used to trim, assemble, align and concatenate the obtained DNA sequences.

### Statistics

The statistical power of microsatellite data to detect true population differentiation and α-error probability were assessed in POWSIM v4.1 [9]. The simulation was performed for effective population size *Ne* =1000 with several numbers of generations (t) to obtain a specific *F_ST_* (t=10 for *F_ST_* =0.001, t= 25 for *F_ST_* =0.0025, t=51 for *F_ST_* =0.005, t=102 for *F_ST_* =0.01, t=10 for *F_ST_* =0.001, and finally t=0 for *F_ST_* =0 to detect α error probability). One thousand replications were run for each level of differentiation. The analyses were conducted using 1000 dememorisations, 100 batches and 1000 iterations per batch.

STRUCTURE v2.3.4 [10] was used to determine the probable number of distinct populations (K) and individuals were assigned to populations using a Bayesian assignment approach. Parameters were set to 5,000,000 MCMC iterations with a burn-in of 500,000 values of K between one and ten, with a series of ten independent replicates for each K value, assuming an admixture model and correlated allele frequencies across the populations, both with and without introducing a priori sampling location.

**Table C. Primers used to amplify the mtDNA genes.**

| **Gene** | **Primer ID** | **Sequence (5' to 3')** | **Annealing Temperature (˚C)** | **Extension time (sec**) | **Reference** | **Taxa** |
| --- | --- | --- | --- | --- | --- | --- |
| *16S rRNA* | ^¥^16S-F | CGCCTGTTTATCAAAAACAT | 57 to 50* | 60 | [7] | Universal |
|  | ^¥^16S-R | CCGGTCTGAACTCAGATCACGT |  |  | [7] | Universal |
| *CytB* | ^¥^7044-F | ACYDGTGGCCTTCAARGCC | 50 |  | This study | Nudipleura |
|  | ^¥^7686-R | ACCATTCAGGTATGAGGATG |  |  | This study | *P. maculata* |
|  | ^¥^151F-MOD | TGRGGWGCWACAGTAATTACTAA | 45 | 80 | [8] | Nudipleura |
|  | ^¥^270R-MOD | AAYAARAARTATCAYTCAGGTTG |  |  | [8] | Nudipleura |
|  | ^¥^*cytB* F | CATGCTAATGGGGCTTCYTTRT | 53 | 80 | This study | *P. maculata* |
|  | ^¥^*cytB* R | ATWGASCGWAAAATRGCATAAGC |  |  | This study | *P. maculata* |
|  | ^¥^8077-F | CTTTCCGACATTACTTGGAGATCC | 53 | 80 | This study | *P. maculata* |
|  | ^¥^8926-R | CCWAVTAGAAMTACTATAGCATG |  |  | This study | Nudipleura |
|  | ^†a^var268-F | TCGTCAAACTAGACCAGCGG | 61.5 to 53* | 90 | This study | *P. maculata* |
|  | ^†a^var1128-R | ACAAGAGCAATAACTCCCCC |  |  | This study | *P. maculata* |
|  | ^†b^8077-F | CTTTCCGACATTACTTGGAGATCC | 61.5 to 53* | 90 | This study | *P. maculata* |
|  | ^†b^var1584-R | TGCCTTCAAAATCCCACTGA |  |  | This study | *P. maculata* |
| *COI* | ^¥^dsgn-F | GCGWTGAYTATTTTCDACAAAYC | 50 | 80 | This study | Nudipleura |
|  | ^¥^universal-R | TAAACTTCAGGGTGACCAAAAATCA |  |  | [6] | Universal |
|  | ^¥^cons2203-F | GGAATAGATGATGATACTCG | 53 | 60 | This study | *P. maculata* |
|  | ^¥^cons2834-R | GTATTACTGTGAAAATCTAAAGG |  |  | This study | *P. maculata* |
|  | ^¥^cons1877-F | GATCTGTTTTAGTGACTGC | 53 | 60 | This study | *P. maculata* |
|  | ^¥^cons2834-R | GTATTACTGTGAAAATCTAAAGG |  |  | This study | *P. maculata* |
|  | ^†a^dsgn-F | GCGWTGAYTATTTTCDACAAAYC | 53 | 80 | This study | *P. maculata* |
|  | ^†a^cons688-R | GTTGGTAAAGAATAGGATCACCTC |  |  | This study | *P. maculata* |
|  | ^†b^var422-F | CGGGGGCGTCTTCTCTACTA | 61.5 to 53* | 90 | This study | *P. maculata* |
|  | ^†b^var1278-R | CCGTCGAGGCATACCAGAAA |  |  | This study | *P. maculata* |

*Touchdown thermocycling conditions. † Primer pairs used to genotype all samples. ^¥^ Primer pairs used to amplify the*16S rRNA*, *cytB* and *COI* first in seven *P. maculata* individual from six localities. Primer pairs used to amplify ^a^ 5’ half and ^b^ 3’ half of the gene locus.

## Supplementary results

### Genetic diversity

**Table D. Summary genetic diversity statistics for the 12 microsatellites and 5 populations of *Pleurobranchaea maculata*.**

| **Pop** | **Locus** | ***N*** | ***Na*** | ***A_R_*** | ***PA_R_*** | ***H_o_*** | ***H_e_*** | ***F_IS_*** | ***P-HWE*** |
| --- | --- | --- | --- | --- | --- | --- | --- | --- | --- |
| **Ti Point** |  |  |  |  |  |  |  |  |  |
|  | ***Pm01*** | 20 | 9 | 8.68 | 0.00 | 0.800 | 0.751 | -0.039 | 0,496 |
|  | ***Pm02*** | 20 | 8 | 7.78 | 0.12 | 0.600 | 0.706 | 0.175 | 0,108 |
|  | ***Pm07*** | 20 | 6 | 5.89 | 0.22 | 0.750 | 0.756 | 0.034 | 0,478 |
|  | ***Pm08*** | 20 | 5 | 4.80 | 0.90 | 0.650 | 0.678 | 0.066 | 0,410 |
|  | ***Pm09*** | 20 | 9 | 8.59 | 0.73 | 0.800 | 0.738 | -0.059 | 0,425 |
|  | ***Pm10*** | 20 | 5 | 4.90 | 0.36 | 0.800 | 0.685 | -0.143 | 0,213 |
|  | ***Pm11*** | 20 | 8 | 7.79 | 0.25 | 0.900 | 0.809 | -0.087 | 0,301 |
|  | ***Pm13*** | 20 | 5 | 4.80 | 0.00 | 0.600 | 0.530 | -0.107 | 0,367 |
|  | ***Pm17*** | 20 | 5 | 4.80 | 0.40 | 0.750 | 0.585 | -0.258 | 0,092 |
|  | ***Pm19*** | 20 | 3 | 3.00 | 0.00 | 0.400 | 0.536 | 0.278 | 0,080 |
|  | ***Pm20*** | 20 | 2 | 2.00 | 0.00 | 0.400 | 0.495 | 0.216 | 0,300 |
|  | ***Pm23*** | 20 | 8 | 7.88 | 0.41 | 0.700 | 0.688 | 0.007 | 0,596 |
|  | **Mean** | 20 | 6.083 | 5.91 | 0.28 | 0.679 | 0.663 | 0.001 | 0,490 |
|  | **SE** | 0 | 2.314 | 0.64 | 0.09 | 0.045 | 0.029 | - | - |
| **Auckland** |  |  |  |  |  |  |  |  |  |
|  | ***Pm01*** | 30 | 12 | 10.20 | 0.03 | 0.833 | 0.767 | -0.070 | 0,275 |
|  | ***Pm02*** | 30 | 7 | 6.28 | 0.01 | 0.833 | 0.767 | 0.067 | 0,320 |
|  | ***Pm07*** | 30 | 7 | 6.17 | 0.20 | 0.833 | 0.767 | 0.108 | 0,186 |
|  | ***Pm08*** | 30 | 4 | 3.94 | 0.00 | 0.833 | 0.767 | -0.047 | 0,436 |
|  | ***Pm09*** | 30 | 9 | 8.04 | 0.31 | 0.833 | 0.767 | 0.029 | 0,456 |
|  | ***Pm10*** | 30 | 6 | 5.44 | 0.06 | 0.833 | 0.767 | -0.039 | 0,453 |
|  | ***Pm11*** | 30 | 8 | 7.38 | 0.00 | 0.833 | 0.767 | -0.084 | 0,224 |
|  | ***Pm13*** | 30 | 6 | 5.29 | 0.38 | 0.833 | 0.767 | -0.005 | 0,591 |
|  | ***Pm17*** | 30 | 4 | 3.20 | 0.07 | 0.833 | 0.767 | 0.156 | 0,227 |
|  | ***Pm19*** | 30 | 4 | 3.60 | 0.04 | 0.833 | 0.767 | -0.152 | 0,229 |
|  | ***Pm20*** | 30 | 3 | 2.60 | 0.60 | 0.833 | 0.767 | -0.074 | 0,270 |
|  | ***Pm23*** | 30 | 10 | 8.06 | 1.80 | 0.833 | 0.767 | -0.136 | 0,314 |
|  | **Mean** | 30 | 6.667 | 5.85 | 0.29 | 0.681 | 0.656 | -0.021 | 0,281 |
|  | **SE** | 0 | 2.741 | 0.66 | 0.15 | 0.037 | 0.032 | - | - |
| **Tauranga** |  |  |  |  |  |  |  |  |  |
|  | ***Pm01*** | 33 | 13 | 10.54 | 0.33 | 0.879 | 0.667 | -0.069 | 0,248 |
|  | ***Pm02*** | 33 | 8 | 6.60 | 0.36 | 0.810 | 0.719 | 0.088 | 0,242 |
|  | ***Pm07*** | 33 | 6 | 5.50 | 0.13 | 0.879 | 0.667 | -0.199 | 0,027 |
|  | ***Pm08*** | 33 | 5 | 4.53 | 0.00 | 0.810 | 0.719 | -0.134 | 0,168 |
|  | ***Pm09*** | 33 | 11 | 9.65 | 0.28 | 0.879 | 0.667 | -0.067 | 0,270 |
|  | ***Pm10*** | 33 | 5 | 4.54 | 0.00 | 0.810 | 0.719 | 0.005 | 0,553 |
|  | ***Pm11*** | 33 | 10 | 8.51 | 0.03 | 0.879 | 0.667 | -0.053 | 0,336 |
|  | ***Pm13*** | 33 | 5 | 4.09 | 0.09 | 0.810 | 0.719 | -0.085 | 0,317 |
|  | ***Pm17*** | 33 | 4 | 3.34 | 0.56 | 0.879 | 0.667 | 0.170 | 0,178 |
|  | ***Pm19*** | 33 | 5 | 4.59 | 0.14 | 0.810 | 0.719 | 0.019 | 0,513 |
|  | ***Pm20*** | 33 | 2 | 2.00 | 0.00 | 0.879 | 0.667 | -0.103 | 0,398 |
|  | ***Pm23*** | 33 | 8 | 6.78 | 0.60 | 0.810 | 0.719 | -0.067 | 0,328 |
|  | **Mean** | 33 | 6.833 | 5.89 | 0.21 | 0.697 | 0.665 | -0.032 | 0,134 |
|  | **SE** | 0 | 3.215 | 0.75 | 0.06 | 0.045 | 0.032 | - | - |
| **Wellington** |  |  |  |  |  |  |  |  |  |
|  | ***Pm01*** | 18 | 13 | 13.00 | 1.65 | 0.833 | 0.667 | 0.097 | 0,169 |
|  | ***Pm02*** | 18 | 6 | 6.00 | 0.00 | 0.895 | 0.731 | 0.117 | 0,266 |
|  | ***Pm07*** | 18 | 5 | 5.00 | 0.00 | 0.833 | 0.667 | 0.124 | 0,309 |
|  | ***Pm08*** | 18 | 5 | 5.00 | 0.45 | 0.895 | 0.731 | -0.148 | 0,264 |
|  | ***Pm09*** | 18 | 7 | 7.00 | 1.00 | 0.833 | 0.667 | 0.146 | 0,180 |
|  | ***Pm10*** | 18 | 5 | 5.00 | 0.00 | 0.895 | 0.731 | -0.073 | 0,426 |
|  | ***Pm11*** | 18 | 9 | 9.00 | 0.38 | 0.833 | 0.667 | 0.065 | 0,367 |
|  | ***Pm13*** | 18 | 5 | 5.00 | 0.03 | 0.895 | 0.731 | 0.103 | 0,354 |
|  | ***Pm17*** | 18 | 4 | 4.00 | 0.60 | 0.833 | 0.667 | 0.038 | 0,515 |
|  | ***Pm19*** | 18 | 2 | 2.00 | 0.00 | 0.895 | 0.731 | 0.029 | 0,651 |
|  | ***Pm20*** | 18 | 2 | 2.00 | 0.00 | 0.833 | 0.667 | -0.214 | 0,484 |
|  | ***Pm23*** | 18 | 9 | 9.00 | 1.58 | 0.895 | 0.731 | 0.089 | 0,301 |
|  | **Mean** | 18 | 6 | 6.00 | 0.48 | 0.606 | 0.618 | 0.047 | 0,127 |
|  | **SE** | 0 | 3.133 | 0.90 | 0.18 | 0.045 | 0.051 | - | - |
| **Nelson** |  |  |  |  |  |  |  |  |  |
|  | ***Pm01*** | 45 | 21 | 15.25 | 4.14 | 0.867 | 0.756 | 0.057 | 0,146 |
|  | ***Pm02*** | 45 | 8 | 6.55 | 0.18 | 0.909 | 0.782 | 0.045 | 0,332 |
|  | ***Pm07*** | 45 | 10 | 6.87 | 1.29 | 0.867 | 0.756 | -0.177 | 0,014 |
|  | ***Pm08*** | 45 | 3 | 3.00 | 0.00 | 0.909 | 0.782 | -0.148 | 0,121 |
|  | ***Pm09*** | 45 | 11 | 7.11 | 0.92 | 0.867 | 0.756 | -0.018 | 0,498 |
|  | ***Pm10*** | 45 | 5 | 4.69 | 0.00 | 0.909 | 0.782 | -0.029 | 0,455 |
|  | ***Pm11*** | 45 | 9 | 7.45 | 0.42 | 0.867 | 0.756 | 0.005 | 0,545 |
|  | ***Pm13*** | 45 | 5 | 3.80 | 0.80 | 0.909 | 0.782 | 0.127 | 0,156 |
|  | ***Pm17*** | 45 | 6 | 4.15 | 0.40 | 0.867 | 0.756 | -0.136 | 0,155 |
|  | ***Pm19*** | 44 | 4 | 3.62 | 0.25 | 0.909 | 0.782 | -0.253 | 0,015 |
|  | ***Pm20*** | 44 | 4 | 2.81 | 0.82 | 0.867 | 0.756 | -0.093 | 0,567 |
|  | ***Pm23*** | 43 | 7 | 5.82 | 0.00 | 0.909 | 0.782 | -0.044 | 0,375 |
|  | **Mean** | 44.667 | 7.750 | 5.93 | 0.77 | 0.641 | 0.609 | -0.041 | 0,044 |
|  | **SE** | 0.651 | 4.883 | 0.97 | 0.33 | 0.056 | 0.057 | - | - |

*N*: Sample size. *Na*: Number of alleles. *A_R_* and *PA_R_*: Allelic richness and private richness, respectively based on 18 diploid individuals. *H_o_*: observed heterozygosity. *H_e_*: unbiased expected heterozygosity. *F_IS_*: inbreeding coefficient [11]. *P-HWE*: *P* values for deviations from *HWE*, a Significant deviation from *HWE* (P<0.05).

**Table E. Allele frequencies at 12 microsatellite loci by population.**

| **Locus** | **Allele (bp)** | **Ti Point** | **Auckland** | **Tauranga** | **Wellington** | **Nelson** |
| --- | --- | --- | --- | --- | --- | --- |
| ***Pm01*** | **Sample size (*N*)** | 20 | 30 | 33 | 18 | 45 |
|  | **108** | 0.000 | 0.017 | 0.000 | 0.000 | 0.133 |
|  | **112** | 0.025 | 0.050 | 0.045 | 0.000 | 0.000 |
|  | **116** | 0.425 | 0.433 | 0.348 | 0.139 | 0.011 |
|  | **120** | 0.075 | 0.083 | 0.152 | 0.028 | 0.056 |
|  | **124** | 0.200 | 0.150 | 0.182 | 0.167 | 0.011 |
|  | **128** | 0.050 | 0.083 | 0.045 | 0.056 | 0.033 |
|  | **132** | 0.000 | 0.033 | 0.015 | 0.056 | 0.011 |
|  | **136** | 0.000 | 0.000 | 0.015 | 0.083 | 0.056 |
|  | **140** | 0.025 | 0.033 | 0.045 | 0.056 | 0.167 |
|  | **144** | 0.050 | 0.050 | 0.030 | 0.111 | 0.156 |
|  | **148** | 0.125 | 0.017 | 0.045 | 0.139 | 0.056 |
|  | **152** | 0.000 | 0.000 | 0.000 | 0.083 | 0.022 |
|  | **156** | 0.000 | 0.017 | 0.015 | 0.000 | 0.056 |
|  | **160** | 0.025 | 0.033 | 0.000 | 0.028 | 0.011 |
|  | **164** | 0.000 | 0.000 | 0.045 | 0.000 | 0.044 |
|  | **168** | 0.000 | 0.000 | 0.015 | 0.000 | 0.022 |
|  | **172** | 0.000 | 0.000 | 0.000 | 0.028 | 0.000 |
|  | **176** | 0.000 | 0.000 | 0.000 | 0.000 | 0.044 |
|  | **180** | 0.000 | 0.000 | 0.000 | 0.028 | 0.033 |
|  | **184** | 0.000 | 0.000 | 0.000 | 0.000 | 0.022 |
|  | **192** | 0.000 | 0.000 | 0.000 | 0.000 | 0.022 |
|  | **196** | 0.000 | 0.000 | 0.000 | 0.000 | 0.022 |
|  | **208** | 0.000 | 0.000 | 0.000 | 0.000 | 0.011 |
| ***Pm02*** | ***N*** | 20 | 30 | 33 | 18 | 45 |
|  | **105** | 0.075 | 0.033 | 0.045 | 0.028 | 0.078 |
|  | **109** | 0.050 | 0.000 | 0.030 | 0.056 | 0.033 |
|  | **113** | 0.475 | 0.467 | 0.424 | 0.306 | 0.244 |
|  | **117** | 0.225 | 0.233 | 0.212 | 0.361 | 0.289 |
|  | **121** | 0.075 | 0.100 | 0.227 | 0.194 | 0.244 |
|  | **125** | 0.025 | 0.117 | 0.000 | 0.056 | 0.089 |
|  | **129** | 0.025 | 0.017 | 0.030 | 0.000 | 0.000 |
|  | **133** | 0.050 | 0.033 | 0.015 | 0.000 | 0.011 |
|  | **137** | 0.000 | 0.000 | 0.015 | 0.000 | 0.011 |
| ***Pm07*** | ***N*** | 20 | 30 | 33 | 18 | 45 |
|  | **128** | 0.000 | 0.017 | 0.015 | 0.000 | 0.011 |
|  | **132** | 0.250 | 0.217 | 0.212 | 0.306 | 0.222 |
|  | **136** | 0.350 | 0.417 | 0.470 | 0.528 | 0.433 |
|  | **140** | 0.200 | 0.183 | 0.091 | 0.028 | 0.100 |
|  | **144** | 0.125 | 0.083 | 0.152 | 0.111 | 0.144 |
|  | **148** | 0.050 | 0.067 | 0.061 | 0.028 | 0.044 |
|  | **152** | 0.025 | 0.017 | 0.000 | 0.000 | 0.011 |
|  | **156** | 0.000 | 0.000 | 0.000 | 0.000 | 0.011 |
|  | **160** | 0.000 | 0.000 | 0.000 | 0.000 | 0.011 |
|  | **164** | 0.000 | 0.000 | 0.000 | 0.000 | 0.011 |
| ***Pm08*** | ***N*** | 20 | 30 | 33 | 18 | 45 |
|  | **141** | 0.200 | 0.267 | 0.106 | 0.028 | 0.000 |
|  | **145** | 0.025 | 0.050 | 0.076 | 0.556 | 0.533 |
|  | **149** | 0.375 | 0.300 | 0.379 | 0.194 | 0.156 |
|  | **153** | 0.375 | 0.383 | 0.424 | 0.194 | 0.311 |
|  | **157** | 0.000 | 0.000 | 0.015 | 0.028 | 0.000 |
|  | **165** | 0.025 | 0.000 | 0.000 | 0.000 | 0.000 |
| ***Pm09*** | ***N*** | 20 | 30 | 33 | 18 | 45 |
|  | **91** | 0.000 | 0.000 | 0.000 | 0.000 | 0.011 |
|  | **97** | 0.000 | 0.050 | 0.061 | 0.000 | 0.000 |
|  | **100** | 0.000 | 0.000 | 0.000 | 0.000 | 0.011 |
|  | **103** | 0.450 | 0.500 | 0.455 | 0.500 | 0.389 |
|  | **106** | 0.150 | 0.083 | 0.061 | 0.028 | 0.011 |
|  | **109** | 0.000 | 0.000 | 0.030 | 0.111 | 0.111 |
|  | **115** | 0.025 | 0.000 | 0.000 | 0.000 | 0.033 |
|  | **118** | 0.050 | 0.067 | 0.061 | 0.000 | 0.011 |
|  | **121** | 0.150 | 0.083 | 0.045 | 0.167 | 0.056 |
|  | **124** | 0.025 | 0.017 | 0.015 | 0.056 | 0.344 |
|  | **127** | 0.025 | 0.000 | 0.076 | 0.111 | 0.011 |
|  | **130** | 0.000 | 0.000 | 0.000 | 0.028 | 0.000 |
|  | **133** | 0.025 | 0.000 | 0.000 | 0.000 | 0.011 |
|  | **136** | 0.100 | 0.050 | 0.076 | 0.000 | 0.000 |
|  | **139** | 0.000 | 0.133 | 0.106 | 0.000 | 0.000 |
|  | **142** | 0.000 | 0.017 | 0.015 | 0.000 | 0.000 |
| ***Pm10*** | ***N*** | 20 | 30 | 33 | 18 | 45 |
|  | **107** | 0.025 | 0.017 | 0.000 | 0.000 | 0.000 |
|  | **110** | 0.475 | 0.400 | 0.379 | 0.111 | 0.056 |
|  | **113** | 0.150 | 0.200 | 0.288 | 0.444 | 0.444 |
|  | **116** | 0.225 | 0.250 | 0.197 | 0.361 | 0.389 |
|  | **119** | 0.000 | 0.100 | 0.121 | 0.056 | 0.078 |
|  | **122** | 0.125 | 0.033 | 0.015 | 0.028 | 0.033 |
| ***Pm11*** | ***N*** | 20 | 30 | 33 | 18 | 45 |
|  | **157** | 0.000 | 0.000 | 0.030 | 0.028 | 0.000 |
|  | **160** | 0.125 | 0.150 | 0.167 | 0.028 | 0.078 |
|  | **163** | 0.225 | 0.200 | 0.242 | 0.306 | 0.467 |
|  | **166** | 0.300 | 0.250 | 0.227 | 0.111 | 0.067 |
|  | **169** | 0.125 | 0.217 | 0.182 | 0.111 | 0.133 |
|  | **172** | 0.125 | 0.083 | 0.045 | 0.083 | 0.056 |
|  | **175** | 0.025 | 0.033 | 0.045 | 0.056 | 0.033 |
|  | **178** | 0.050 | 0.050 | 0.030 | 0.250 | 0.144 |
|  | **181** | 0.025 | 0.000 | 0.015 | 0.000 | 0.011 |
|  | **184** | 0.000 | 0.017 | 0.015 | 0.028 | 0.000 |
|  | **187** | 0.000 | 0.000 | 0.000 | 0.000 | 0.011 |
| ***Pm13*** | ***N*** | 20 | 30 | 33 | 18 | 45 |
|  | **103** | 0.000 | 0.000 | 0.000 | 0.000 | 0.011 |
|  | **118** | 0.000 | 0.000 | 0.000 | 0.000 | 0.011 |
|  | **121** | 0.025 | 0.033 | 0.000 | 0.028 | 0.000 |
|  | **124** | 0.000 | 0.033 | 0.015 | 0.000 | 0.000 |
|  | **127** | 0.125 | 0.150 | 0.197 | 0.111 | 0.144 |
|  | **130** | 0.650 | 0.600 | 0.591 | 0.556 | 0.644 |
|  | **133** | 0.175 | 0.167 | 0.182 | 0.278 | 0.189 |
|  | **136** | 0.025 | 0.017 | 0.015 | 0.028 | 0.000 |
| ***Pm17*** | ***N*** | 20 | 30 | 33 | 18 | 45 |
|  | **155** | 0.025 | 0.017 | 0.030 | 0.000 | 0.011 |
|  | **167** | 0.325 | 0.300 | 0.333 | 0.083 | 0.067 |
|  | **169** | 0.000 | 0.000 | 0.000 | 0.028 | 0.011 |
|  | **171** | 0.025 | 0.017 | 0.000 | 0.000 | 0.000 |
|  | **173** | 0.000 | 0.000 | 0.015 | 0.000 | 0.000 |
|  | **175** | 0.550 | 0.667 | 0.621 | 0.806 | 0.811 |
|  | **179** | 0.075 | 0.000 | 0.000 | 0.083 | 0.089 |
|  | **187** | 0.000 | 0.000 | 0.000 | 0.000 | 0.011 |
| ***Pm19*** | ***N*** | 20 | 30 | 33 | 18 | 44 |
|  | **169** | 0.000 | 0.000 | 0.030 | 0.000 | 0.068 |
|  | **172** | 0.150 | 0.100 | 0.152 | 0.333 | 0.216 |
|  | **175** | 0.625 | 0.600 | 0.636 | 0.667 | 0.693 |
|  | **178** | 0.225 | 0.283 | 0.152 | 0.000 | 0.000 |
|  | **181** | 0.000 | 0.017 | 0.030 | 0.000 | 0.023 |
| ***Pm20*** | ***N*** | 20 | 30 | 33 | 18 | 44 |
|  | **114** | 0.000 | 0.000 | 0.000 | 0.000 | 0.011 |
|  | **120** | 0.550 | 0.617 | 0.530 | 0.194 | 0.091 |
|  | **126** | 0.450 | 0.367 | 0.470 | 0.806 | 0.886 |
|  | **129** | 0.000 | 0.017 | 0.000 | 0.000 | 0.000 |
|  | **132** | 0.000 | 0.000 | 0.000 | 0.000 | 0.011 |
| ***Pm23*** | ***N*** | 20 | 30 | 33 | 18 | 43 |
|  | **156** | 0.075 | 0.083 | 0.091 | 0.500 | 0.512 |
|  | **158** | 0.000 | 0.000 | 0.000 | 0.028 | 0.012 |
|  | **160** | 0.050 | 0.033 | 0.030 | 0.056 | 0.140 |
|  | **162** | 0.050 | 0.033 | 0.045 | 0.111 | 0.093 |
|  | **164** | 0.525 | 0.567 | 0.515 | 0.028 | 0.116 |
|  | **166** | 0.100 | 0.150 | 0.212 | 0.083 | 0.116 |
|  | **168** | 0.100 | 0.067 | 0.076 | 0.056 | 0.012 |
|  | **170** | 0.075 | 0.017 | 0.000 | 0.111 | 0.000 |
|  | **172** | 0.025 | 0.000 | 0.015 | 0.000 | 0.000 |
|  | **174** | 0.000 | 0.017 | 0.000 | 0.000 | 0.000 |
|  | **176** | 0.000 | 0.017 | 0.000 | 0.000 | 0.000 |
|  | **178** | 0.000 | 0.000 | 0.000 | 0.028 | 0.000 |
|  | **182** | 0.000 | 0.017 | 0.000 | 0.000 | 0.000 |
|  | **184** | 0.000 | 0.000 | 0.015 | 0.000 | 0.000 |

| **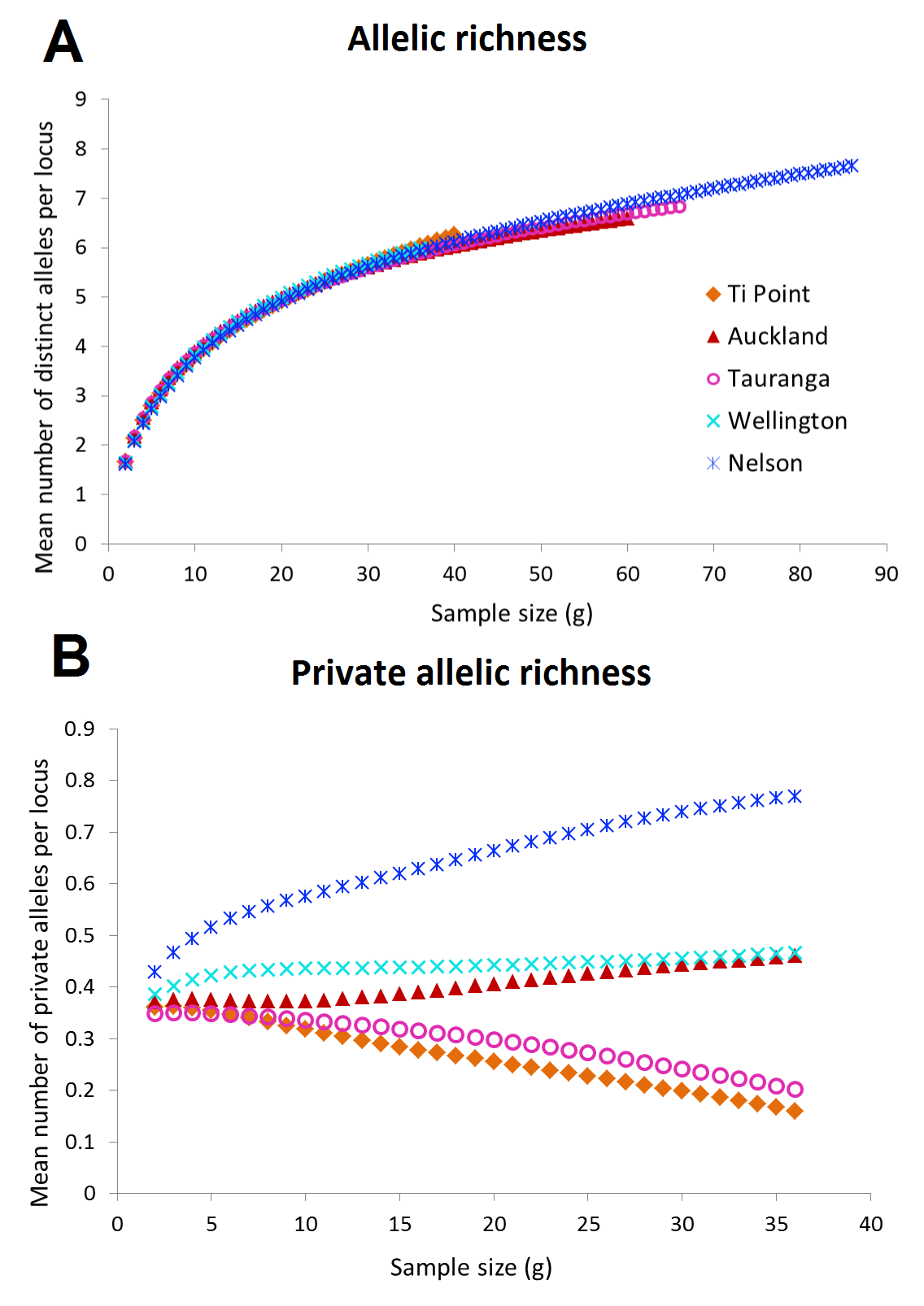****Figure A. Allele rarefaction curves for the microsatellite data.** |
| --- |

**Table F. Results of the linkage equilibrium test for 12 microsatellite markers.** The calculations were performed for each population and across the populations. *P* values were estimated by 6600 permutations. The *P*-value for the 5% nominal level is 0.000152 after Bonferroni correction.

| Loci | Ti Point | Auckland | Tauranga | Wellington | Nelson | Overall |
| --- | --- | --- | --- | --- | --- | --- |
| *Pm01 X Pm02* | 0.81924 | 0.98515 | 0.72652 | 1.0000 | 0.31399 | 0.86106 |
| *Pm01 X Pm07* | 0.87697 | 0.72152 | 0.40970 | 0.30455 | 1.00000 | 0.68409 |
| *Pm01 X Pm08* | 0.91530 | 0.21091 | 0.79197 | 1.00000 | 0.67792 | 0.64470 |
| *Pm01 X Pm09* | 0.63227 | 0.83258 | 0.00712 | 0.20924 | 1.0000 | 0.15894 |
| *Pm01 X Pm10* | 0.86636 | 0.98682 | 0.69621 | 0.38303 | 0.69394 | 0.87106 |
| *Pm01 X Pm11* | 0.67030 | 0.53621 | 0.18682 | 1.0000 | 0.24057 | 0.12500 |
| *Pm01 X Pm13* | 0.57409 | 0.38455 | 0.88758 | 1.0000 | 0.89792 | 0.81909 |
| *Pm01 X Pm17* | 0.93909 | 0.20167 | 0.58818 | 0.72318 | 0.01661 | 0.11561 |
| *Pm01 X Pm20* | 0.88712 | 0.59167 | 0.25742 | 0.74697 | 0.64158 | 0.58985 |
| *Pm01 X Pm23* | 0.16152 | 0.60909 | 0.70652 | 1.00000 | 0.46336 | 0.40985 |
| *Pm01 X Pm19* | 0.99303 | 0.76742 | 0.35864 | 1.00000 | 0.34141 | 0.76121 |
| *Pm02 X Pm07* | 0.96742 | 0.84000 | 0.935 | 0.56803 | 0.30578 | 0.84515 |
| *Pm02 X Pm08* | 0.97136 | 0.52379 | 0.39379 | 0.0303 | 0.94727 | 0.6997 |
| *Pm02 X Pm09* | 0.07712 | 0.14515 | 0.63091 | 0.75333 | 0.62534 | 0.19379 |
| *Pm02 X Pm10* | 0.95242 | 0.43364 | 0.92227 | 0.70621 | 0.73302 | 0.91076 |
| *Pm02 X Pm11* | 0.80182 | 0.88712 | 0.97106 | 0.37455 | 0.28805 | 0.72182 |
| *Pm02 X Pm13* | 0.73909 | 0.68227 | 0.36955 | 0.92682 | 0.22655 | 0.51712 |
| *Pm02 X Pm17* | 0.45636 | 0.53545 | 0.52742 | 0.57909 | 0.0888 | 0.22121 |
| *Pm02 X Pm20* | 0.38515 | 0.89288 | 0.98894 | 0.97879 | 0.81632 | 0.99318 |
| *Pm02 X Pm23* | 0.54379 | 0.75621 | 0.90848 | 1.0000 | 0.89751 | 0.96848 |
| *Pm02 X Pm19* | 0.74939 | 0.84788 | 0.40939 | 0.62955 | 0.66934 | 0.81591 |
| *Pm07 X Pm08* | 0.60606 | 0.45515 | 0.05409 | 0.98894 | 0.47701 | 0.34727 |
| *Pm07 X Pm09* | 0.84015 | 0.73182 | 0.92091 | 0.11515 | 0.45496 | 0.54136 |
| *Pm07 X Pm10* | 0.82379 | 0.84076 | 0.17985 | 0.66015 | 0.87339 | 0.73318 |
| *Pm07 X Pm11* | 0.07788 | 0.93955 | 0.35591 | 1.00000 | 0.45026 | 0.42500 |
| *Pm07 X Pm13* | 0.27030 | 0.32727 | 0.92561 | 0.50909 | 0.59180 | 0.60515 |
| *Pm07 X Pm17* | 0.79879 | 0.16045 | 0.53364 | 0.36879 | 0.45765 | 0.37439 |
| *Pm07 X Pm20* | 0.80000 | 0.39197 | 0.66409 | 1.00000 | 0.07875 | 0.45106 |
| *Pm07 X Pm23* | 0.92894 | 0.10667 | 0.23833 | 0.72788 | 0.96035 | 0.63106 |
| *Pm07 X Pm19* | 0.20318 | 0.15273 | 0.61636 | 0.59364 | 0.90050 | 0.53152 |
| *Pm08 X Pm09* | 1.00000 | 0.70152 | 0.71167 | 0.85561 | 0.51109 | 0.84545 |
| *Pm08 X Pm10* | 0.54348 | 0.94273 | 0.59061 | 0.94500 | 0.39336 | 0.84621 |
| *Pm08 X Pm11* | 1.00000 | 0.54333 | 0.00121 | 0.25394 | 0.70118 | 0.13955 |
| *Pm08 X Pm13* | 1.00000 | 0.21485 | 0.19121 | 0.06667 | 0.55496 | 0.30712 |
| *Pm08 X Pm17* | 0.77242 | 0.53833 | 0.71455 | 0.27606 | 0.56631 | 0.6597 |
| *Pm08 X Pm20* | 0.00879 | 0.02636 | 0.67288 | 0.69576 | 0.12984 | 0.01409 |
| *Pm08 X Pm23* | 0.73030 | 0.47788 | 0.47530 | 1.0000 | 0.69422 | 0.80939 |
| *Pm08 X Pm19* | 0.89939 | 0.22409 | 0.98470 | 0.13197 | 0.95692 | 0.91364 |
| *Pm09 X Pm10* | 0.39606 | 0.51591 | 0.84591 | 0.47470 | 0.08904 | 0.18091 |
| *Pm09 X Pm11* | 0.60424 | 0.82742 | 0.79470 | 1.00000 | 0.95592 | 0.95712 |
| *Pm09 X Pm13* | 0.84879 | 0.52227 | 0.67909 | 0.35212 | 0.08052 | 0.25242 |
| *Pm09 X Pm17* | 0.99303 | 0.68667 | 0.59333 | 0.07121 | 0.09781 | 0.31076 |
| *Pm09 X Pm20* | 0.75864 | 0.37939 | 0.95409 | 0.24833 | 0.70624 | 0.77818 |
| *Pm09 X Pm23* | 0.09667 | 0.71636 | 0.35985 | 0.19970 | 0.02113 | 0.02288 |
| *Pm09 X Pm19* | 0.84000 | 0.90455 | 0.11394 | 0.35348 | 0.58146 | 0.53424 |
| *Pm10 X Pm11* | 0.82379 | 0.98500 | 1.00000 | 1.00000 | 0.31481 | 0.95439 |
| *Pm10 X Pm13* | 0.77091 | 0.05773 | 0.22424 | 0.72167 | 0.25988 | 0.11061 |
| *Pm10 X Pm17* | 0.07955 | 0.49167 | 0.50833 | 0.80409 | 0.71723 | 0.50788 |
| *Pm10 X Pm20* | 0.84061 | 0.65333 | 0.98455 | 0.64727 | 0.79675 | 0.98318 |
| *Pm10 X Pm23* | 0.61242 | 0.85379 | 0.75061 | 0.00515 | 0.54974 | 0.41394 |
| *Pm10 X Pm19* | 0.03318 | 0.05667 | 0.37500 | 0.88758 | 0.35618 | 0.05712 |
| *Pm11 X Pm13* | 0.57909 | 0.99152 | 0.11000 | 0.72485 | 0.99279 | 0.93455 |
| *Pm11 X Pm17* | 0.73439 | 0.59318 | 0.70939 | 0.08424 | 0.1661 | 0.23985 |
| *Pm11 X Pm20* | 0.33591 | 0.75470 | 0.61727 | 1.00000 | 0.44158 | 0.73500 |
| *Pm11 X Pm23* | 0.76773 | 0.64000 | 0.82091 | 1.00000 | 0.82685 | 0.91167 |
| *Pm11 X Pm19* | 0.16106 | 0.31879 | 0.17727 | 0.71742 | 0.31657 | 0.09000 |
| *Pm13 X Pm17* | 0.08076 | 0.33773 | 0.3953 | 0.51788 | 0.86564 | 0.40697 |
| *Pm13 X Pm20* | 0.29424 | 0.57515 | 0.62242 | 0.77136 | 0.46031 | 0.63106 |
| *Pm13 X Pm23* | 0.17121 | 0.18606 | 0.56030 | 0.78379 | 0.56401 | 0.2997 |
| *Pm13 X Pm19* | 0.29303 | 0.08439 | 0.74000 | 0.76545 | 0.23881 | 0.22818 |
| *Pm17 X Pm20* | 0.47697 | 0.07864 | 0.80015 | 0.38015 | 0.59897 | 0.41864 |
| *Pm17 X Pm23* | 0.84591 | 0.57818 | 0.18985 | 0.80076 | 0.48462 | 0.57348 |
| *Pm17 X Pm19* | 0.09712 | 0.12939 | 0.26439 | 0.83970 | 0.23129 | 0.09894 |
| *Pm19 X Pm20* | 0.41848 | 0.47576 | 0.17136 | 1.00000 | 0.51053 | 0.45818 |
| *Pm19 X Pm23* | 0.43697 | 0.29515 | 0.10303 | 0.32955 | 0.20853 | 0.06288 |
| *Pm20 X Pm23* | 0.66167 | 0.75667 | 0.05985 | 0.31667 | 0.70504 | 0.43273 |

### Genetic Structure

**Multivariate approach for mtDNA**

CAP analysis was performed for *COI* but not for *CytB* because PERMANOVA did not reveal significant differences between groups for the latter. A CAP analysis to discriminate the three northern populations was statistically significant (trace=0.293, *P*=0.0038), but the populations varied in their degree of distinctiveness under the CAP model. Specifically, leave-one-out cross-validation had the highest allocation success for TR (69.7%), followed by AKL (50%), but there was little or no identifiability of samples from Ti Point (only 25% correct allocation).

**Table G. Pairwise population differentiation estimates and associated tests across five populations.**

|  | **Microsatellite data** | | | | | | ***COI* data** | | | ***CytB* data*** | |
| --- | --- | --- | --- | --- | --- | --- | --- | --- | --- | --- | --- |
| **Groups** | **Genic** | ***F_ST_*** |  | ***Dest*** | ***t-DM*** | ***t-DCL*** | ***t*** | ***F_ST_*** | ***Θ_ST_*** | ***F_ST_*** | ***Θ_ST_*** |
| TP-AKL | 17.26 | -0.009 |  | -0.019 | 0.699 | 0.766 | 1.585^a^ | 0.014^b^ | 0.024 | 0.050^c^ | 0.028 |
| TP-TR | 29.67 | -0.004 |  | -0.008 | 0.727 | 0.79 | 1.359 | 0.017^b^ | 0.018 | 0.043^b^ | 0.023 |
| TP-WL | Inf^c^ | 0.087^c^ |  | 0.185^c^ | 2.851^c^ | 2.711^c^ | 1.233 | 0.014^a^ | 0.008 | 0.033^c^ | -0.026 |
| TP-NL | Inf^c^ | 0.118^c^ |  | 0.241^c^ | 4.160^c^ | 4.083^c^ | 1.494 | 0.030 ^b^ | 0.029 | 0.040 ^b^ | 0.000 |
| AKL-TR | 20.48 | -0.004 |  | -0.007 | 0.983 | 0.655 | 1.712^a^ | 0.015^c^ | 0.020 | 0.071^c^ | 0.019 |
| AKL-WL | Inf^c^ | 0.095^c^ |  | 0.198^c^ | 2.548^c^ | 2.958^c^ | 1.202 | 0.012^a^ | 0.014 | 0.062^c^ | -0.003 |
| AKL-NL | Inf^c^ | 0.122^c^ |  | 0.246^c^ | 3.677^c^ | 4.693^c^ | 1.283 | 0.028^c^ | 0.005 | 0.068^c^ | -0.001 |
| TR-WL | Inf^c^ | 0.074^c^ |  | 0.153^c^ | 2.541^c^ | 3.035^c^ | 1.403 | 0.016^a^ | 0.017 | 0.056^c^ | 0.012 |
| TR-NL | Inf^c^ | 0.097^c^ |  | 0.194^c^ | 3.725^c^ | 4.661^c^ | 1.076 | 0.031^c^ | 0.007 | 0.061^c^ | 0.026^a^ |
| WL-NL | 53.20^b^ | 0.008^a^ |  | 0.013 | 1.235^a^ | 1.593 ^a^ | 1.326 | 0.029^b^ | 0.020 | 0.053^c^ | -0.014 |

*χ2* values for the homogeneity of allele frequencies in pairwise comparisons tested with the exact *G*-test (Genic). Pseudo *t* statistic (*t*) from pairwise PERMANOVA tests on the basis of either Manhattan (*DM*) or Clonal distances (*DCL*) for microsatellite data, or standardized nucleotide differences for mtDNA data. Other estimators used were fixation index *F_ST_*_,_ Jost’s differentiation (*Dest*) and distance-based *Θ_ST_*. TP: Ti Point, AKL: Auckland, TR: Tauranga, WL: Wellington, NL: Nelson. Significant differentiation: ^a^ *P*<0.05, ^b^ *P*<0.0005, ^c^ *P*≤0.0001. *** Pairwise comparisons using pseudo *t* were not done for *CytB* data because the overall test was not statistically significant.

**Table H. Pairwise population matrix of the *Dest* values at each locus.** *Dest* values below the diagonal. The *P* values calculated with 9999 permutations are shown above the diagonal.

| *Pm01* |  |  |  |  |  |  | *Pm11* |  |  |  |  |  |
| --- | --- | --- | --- | --- | --- | --- | --- | --- | --- | --- | --- | --- |
|  | TP | AKL | TR | WL | NL |  |  | TP | AKL | TR | WL | NL |
| TP | ----- | 0.757 | 0.586 | 0.023 | 0.000 |  | TP | ----- | 0.816 | 0.702 | 0.052 | 0.001 |
| AKL | -0.028 | ----- | 0.525 | 0.002 | 0.000 |  | AKL | -0.049 | ----- | 0.945 | 0.015 | 0.000 |
| TR | -0.017 | -0.009 | ----- | 0.010 | 0.000 |  | TR | -0.036 | -0.053 | ----- | 0.021 | 0.001 |
| WL | 0.205 | 0.302 | 0.222 | ----- | 0.002 |  | WL | 0.157 | 0.181 | 0.167 | ----- | 0.180 |
| NL | 0.793 | 0.787 | 0.730 | 0.374 | ----- |  | NL | 0.235 | 0.245 | 0.188 | 0.031 | ----- |
| *Pm02* |  |  |  |  |  |  | *Pm13* |  |  |  |  |  |
|  | TP | AKL | TR | WL | NL |  |  | TP | AKL | TR | WL | NL |
| TP | ----- | 0.843 | 0.449 | 0.193 | 0.021 |  | TP | ----- | 0.909 | 0.649 | 0.566 | 0.932 |
| AKL | -0.036 | ----- | 0.228 | 0.146 | 0.010 |  | AKL | -0.021 | ----- | 0.893 | 0.568 | 0.788 |
| TR | -0.007 | 0.016 | ----- | 0.284 | 0.063 |  | TR | -0.012 | -0.017 | ----- | 0.488 | 0.654 |
| WL | 0.044 | 0.045 | 0.015 | ----- | 0.743 |  | WL | -0.013 | -0.014 | -0.009 | ----- | 0.469 |
| NL | 0.129 | 0.113 | 0.057 | -0.037 | ----- |  | NL | -0.019 | -0.013 | -0.010 | -0.007 | ----- |
| *Pm07* |  |  |  |  |  |  | Pm*17* |  |  |  |  |  |
|  | TP | AKL | TR | WL | NL |  |  | TP | AKL | TR | WL | NL |
| TP | ----- | 0.936 | 0.353 | 0.171 | 0.568 |  | TP | ----- | 0.327 | 0.506 | 0.007 | 0.000 |
| AKL | -0.054 | ----- | 0.561 | 0.245 | 0.678 |  | AKL | -0.001 | ----- | 0.765 | 0.047 | 0.002 |
| TR | 0.004 | -0.011 | ----- | 0.548 | 0.979 |  | TR | -0.010 | -0.014 | ----- | 0.020 | 0.000 |
| WL | 0.044 | 0.021 | -0.013 | ----- | 0.438 |  | WL | 0.099 | 0.046 | 0.074 | ----- | 1.000 |
| NL | -0.013 | -0.015 | -0.024 | -0.004 | ----- |  | NL | 0.115 | 0.060 | 0.090 | -0.009 | ----- |
| *Pm08* |  |  |  |  |  |  | *Pm19* |  |  |  |  |  |
|  | TP | AKL | TR | WL | NL |  |  | TP | AKL | TR | WL | NL |
| TP | ----- | 0.734 | 0.608 | 0.000 | 0.000 |  | TP | ----- | 0.806 | 0.811 | 0.073 | 0.020 |
| AKL | -0.030 | ----- | 0.164 | 0.000 | 0.000 |  | AKL | -0.020 | ----- | 0.258 | 0.003 | 0.000 |
| TR | -0.019 | 0.023 | ----- | 0.000 | 0.000 |  | TR | -0.020 | 0.007 | ----- | 0.077 | 0.058 |
| WL | 0.510 | 0.498 | 0.426 | ----- | 0.437 |  | WL | 0.056 | 0.120 | 0.037 | ----- | 0.269 |
| NL | 0.466 | 0.449 | 0.368 | -0.004 | ----- |  | NL | 0.047 | 0.098 | 0.020 | 0.003 | ----- |
| *Pm09* |  |  |  |  |  |  | *Pm20* |  |  |  |  |  |
|  | TP | AKL | TR | WL | NL |  |  | TP | AKL | TR | WL | NL |
| TP | ----- | 0.281 | 0.199 | 0.236 | 0.000 |  | TP | ----- | 0.500 | 0.928 | 0.003 | 0.000 |
| AKL | 0.012 | ----- | 0.810 | 0.097 | 0.000 |  | AKL | -0.010 | ----- | 0.282 | 0.000 | 0.000 |
| TR | 0.023 | -0.021 | ----- | 0.129 | 0.000 |  | TR | -0.023 | 0.003 | ----- | 0.002 | 0.000 |
| WL | 0.024 | 0.053 | 0.038 | ----- | 0.007 |  | WL | 0.197 | 0.300 | 0.178 | ----- | 0.148 |
| NL | 0.254 | 0.261 | 0.258 | 0.161 | ----- |  | NL | 0.301 | 0.414 | 0.278 | 0.006 | ----- |
| *Pm10* |  |  |  |  |  |  | *Pm23* |  |  |  |  |  |
|  | TP | AKL | TR | WL | NL |  |  | TP | AKL | TR | WL | NL |
| TP | ----- | 0.390 | 0.102 | 0.000 | 0.000 |  | TP | ----- | 0.874 | 0.561 | 0.000 | 0.000 |
| AKL | 0.000 | ----- | 0.663 | 0.002 | 0.000 |  | AKL | -0.026 | ----- | 0.814 | 0.000 | 0.000 |
| TR | 0.052 | -0.021 | ----- | 0.012 | 0.000 |  | TR | -0.012 | -0.017 | ----- | 0.000 | 0.000 |
| WL | 0.349 | 0.218 | 0.165 | ----- | 0.916 |  | WL | 0.706 | 0.738 | 0.699 | ----- | 0.332 |
| NL | 0.433 | 0.293 | 0.242 | -0.029 | ----- |  | NL | 0.590 | 0.591 | 0.553 | 0.005 | ----- |

TP: Ti Point, AKL: Auckland, TR: Tauranga, WL: Wellington, NL: Nelson.

| 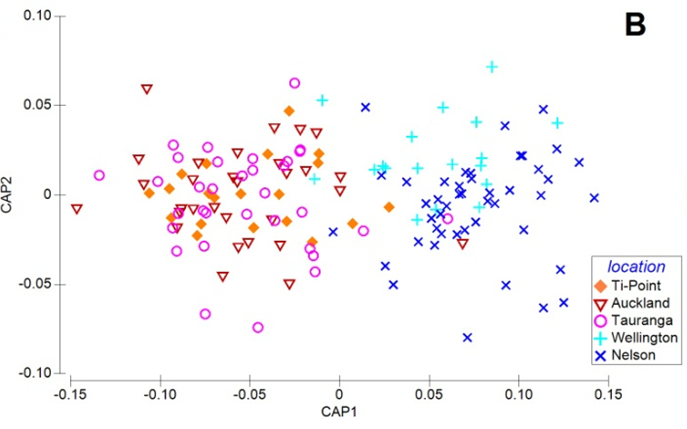  **Figure B. Canonical analysis of principal coordinates of the microsatellite data.** The axes represent the amount of variation between the populations that is explained by the two axes (the number of axes - *m*=13). Each symbol represents a different sampling location. |
| --- |


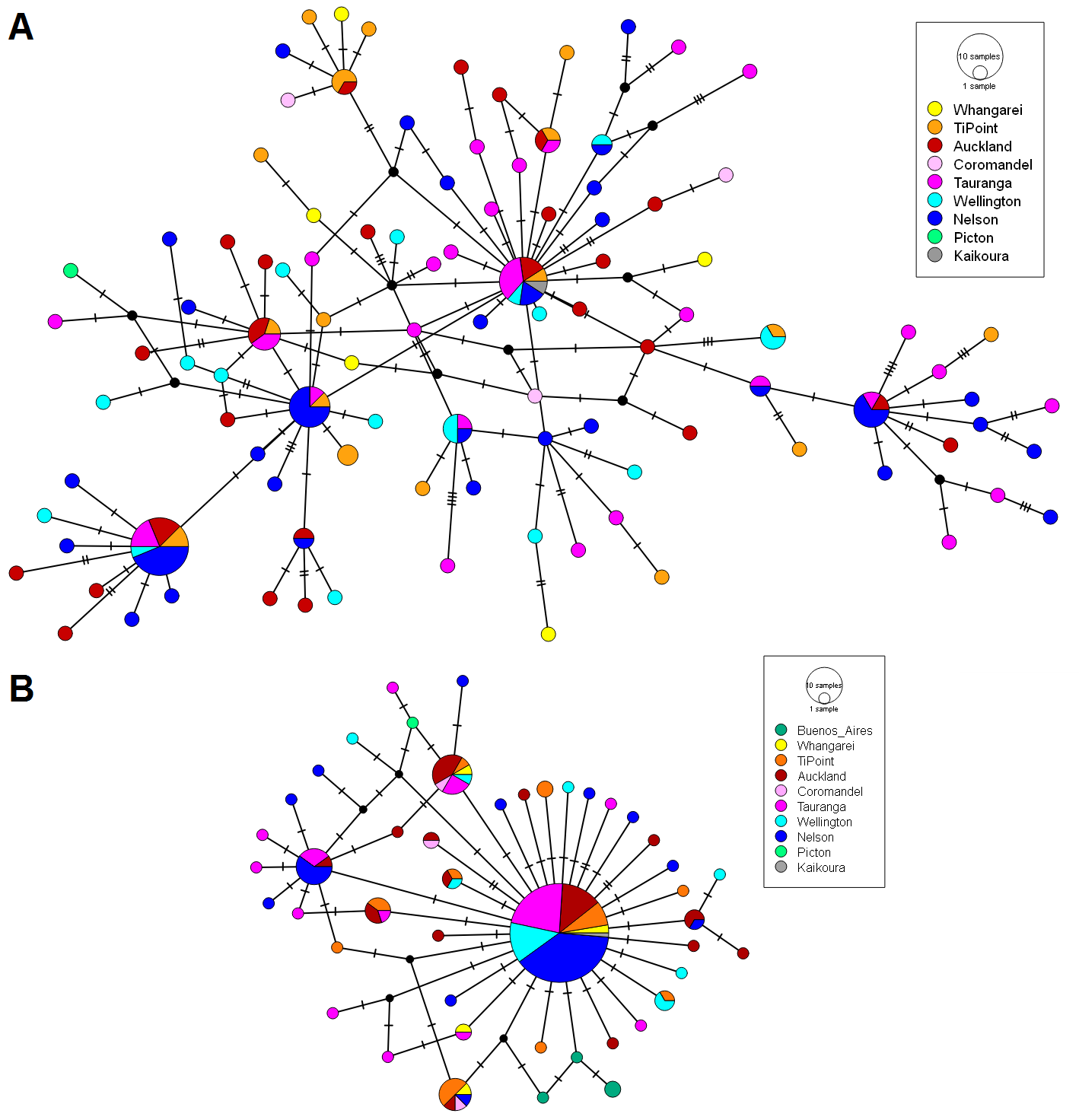


Figure C. Median joining network of the *COI* and *Cytb* haplotypes. (A) 1153 bp of *COI* sequences from 156 New Zealand individuals. (B) 624 bp of *COI* sequences from 156 New Zealand and four individuals from Argentina. The network was coloured according to the sampling locations. The diameters of the circles are proportional to the frequency of the haplotypes. The hashes indicate the mutational steps between the haplotypes. The black nodes represent the imaginary haplotypes necessary to create a bridge between the present haplotypes.

**Table I. Haplotype frequencies for the mtDNA sequences by mere counting in populations.**

| ***COI*** | | | | | | |  | ***CytB*** | | | | | | |
| --- | --- | --- | --- | --- | --- | --- | --- | --- | --- | --- | --- | --- | --- | --- |
|  | **^+^WH** | **TP** | **AK** | **TA** | **WL** | **NL** |  |  | **WH** | **TP** | **AK** | **TA** | **WL** | **NL** |
| **ID** | **5*** | **20** | **30** | **33** | **18** | **45** |  | **ID** | **5** | **20** | **30** | **33** | **18** | **45** |
| 17B | 1 | 0 | 0 | 0 | 0 | 0 |  | 17B | 1 | 0 | 0 | 0 | 0 | 0 |
| 17J | 1 | 0 | 0 | 0 | 0 | 0 |  | 17I | 1 | 0 | 0 | 1 | 0 | 0 |
| 17I | 1 | 0 | 0 | 0 | 0 | 0 |  | 17J | 1 | 0 | 0 | 0 | 0 | 0 |
| 17L | 1 | 0 | 0 | 0 | 0 | 0 |  | 17L | 1 | 2 | 5 | 8 | 1 | 4 |
| 17M | 1 | 0 | 0 | 0 | 0 | 0 |  | 17M | 1 | 1 | 1 | 1 | 0 | 1 |
| 37A | 0 | 1 | 2 | 2 | 0 | 0 |  | 37A | 0 | 2 | 7 | 3 | 3 | 10 |
| 37B | 0 | 1 | 1 | 1 | 0 | 0 |  | 37C | 0 | 1 | 0 | 0 | 0 | 0 |
| 37C | 0 | 2 | 0 | 0 | 0 | 0 |  | 37E | 0 | 2 | 0 | 0 | 1 | 0 |
| 37D | 0 | 1 | 0 | 0 | 0 | 0 |  | 37F | 0 | 1 | 0 | 0 | 0 | 0 |
| 37E | 0 | 1 | 0 | 0 | 0 | 0 |  | 37G | 0 | 1 | 0 | 0 | 0 | 0 |
| 37F | 0 | 2 | 3 | 3 | 1 | 7 |  | 38A | 0 | 1 | 1 | 2 | 0 | 0 |
| 37G | 0 | 2 | 1 | 0 | 0 | 0 |  | 38B | 0 | 1 | 1 | 2 | 0 | 1 |
| 38A | 0 | 1 | 2 | 4 | 1 | 2 |  | 24V | 0 | 1 | 0 | 0 | 0 | 1 |
| 38B | 0 | 1 | 0 | 0 | 0 | 0 |  | 38D | 0 | 1 | 0 | 0 | 0 | 0 |
| 38C | 0 | 1 | 0 | 0 | 0 | 0 |  | 38E | 0 | 1 | 1 | 2 | 3 | 4 |
| 24M | 0 | 1 | 0 | 1 | 0 | 6 |  | 38F | 0 | 2 | 1 | 0 | 0 | 2 |
| 38G | 0 | 1 | 0 | 0 | 0 | 0 |  | 38G | 0 | 1 | 0 | 0 | 0 | 0 |
| 38H | 0 | 1 | 0 | 0 | 0 | 0 |  | 38J | 0 | 1 | 0 | 0 | 0 | 0 |
| 38I | 0 | 1 | 0 | 0 | 0 | 0 |  | 38L | 0 | 1 | 0 | 0 | 0 | 0 |
| 38J | 0 | 1 | 0 | 0 | 2 | 0 |  | NN1 | 0 | 0 | 1 | 0 | 0 | 0 |
| 38K | 0 | 1 | 0 | 0 | 0 | 0 |  | 33B | 0 | 0 | 1 | 0 | 0 | 0 |
| 38L | 0 | 1 | 0 | 0 | 0 | 0 |  | 33D | 0 | 0 | 1 | 0 | 0 | 0 |
| NN1 | 0 | 0 | 1 | 0 | 0 | 0 |  | 40B | 0 | 0 | 3 | 0 | 0 | 0 |
| 33B | 0 | 0 | 1 | 0 | 0 | 0 |  | 41C | 0 | 0 | 1 | 0 | 0 | 0 |
| 33C | 0 | 0 | 1 | 0 | 0 | 0 |  | 41D | 0 | 0 | 1 | 0 | 0 | 0 |
| 33D | 0 | 0 | 1 | 0 | 0 | 0 |  | 41H | 0 | 0 | 1 | 0 | 0 | 0 |
| 33E | 0 | 0 | 1 | 0 | 0 | 0 |  | 42B | 0 | 0 | 1 | 0 | 0 | 0 |
| 36B | 0 | 0 | 1 | 0 | 0 | 0 |  | 42D | 0 | 0 | 1 | 0 | 0 | 0 |
| 36C | 0 | 0 | 1 | 0 | 0 | 0 |  | 42F | 0 | 0 | 1 | 1 | 0 | 0 |
| 40A | 0 | 0 | 1 | 0 | 0 | 0 |  | 44A | 0 | 0 | 1 | 0 | 0 | 0 |
| 45D | 0 | 0 | 1 | 0 | 0 | 1 |  | 34A | 0 | 0 | 0 | 1 | 0 | 0 |
| 41B | 0 | 0 | 1 | 0 | 0 | 0 |  | 34E | 0 | 0 | 0 | 1 | 0 | 0 |
| 41C | 0 | 0 | 1 | 0 | 0 | 0 |  | 34G | 0 | 0 | 0 | 1 | 0 | 0 |
| 41D | 0 | 0 | 1 | 0 | 0 | 0 |  | 34I | 0 | 0 | 0 | 1 | 0 | 0 |
| 41G | 0 | 0 | 1 | 0 | 0 | 0 |  | 34J | 0 | 0 | 0 | 1 | 0 | 0 |
| 41H | 0 | 0 | 1 | 0 | 0 | 0 |  | 47A | 0 | 0 | 0 | 1 | 0 | 0 |
| 42A | 0 | 0 | 1 | 0 | 0 | 0 |  | 47B | 0 | 0 | 0 | 1 | 0 | 0 |
| 42B | 0 | 0 | 1 | 0 | 0 | 0 |  | 47C | 0 | 0 | 0 | 1 | 0 | 0 |
| 42C | 0 | 0 | 1 | 1 | 0 | 4 |  | 47E | 0 | 0 | 0 | 1 | 0 | 0 |
| 42D | 0 | 0 | 1 | 0 | 0 | 0 |  | 32D | 0 | 0 | 0 | 1 | 0 | 0 |
| 42E | 0 | 0 | 1 | 0 | 0 | 0 |  | 32E | 0 | 0 | 0 | 1 | 0 | 1 |
| 42F | 0 | 0 | 1 | 0 | 0 | 0 |  | NI-C | 0 | 0 | 0 | 1 | 0 | 0 |
| 44C | 0 | 0 | 1 | 0 | 0 | 0 |  | NI-E | 0 | 0 | 0 | 1 | 0 | 0 |
| 34A | 0 | 0 | 0 | 1 | 0 | 0 |  | 43B | 0 | 0 | 0 | 0 | 2 | 0 |
| 34E | 0 | 0 | 0 | 1 | 0 | 0 |  | 43C | 0 | 0 | 0 | 0 | 1 | 0 |
| 34F | 0 | 0 | 0 | 1 | 0 | 0 |  | 43D | 0 | 0 | 0 | 0 | 1 | 1 |
| 34G | 0 | 0 | 0 | 1 | 0 | 0 |  | 43E | 0 | 0 | 0 | 0 | 1 | 0 |
| 34H | 0 | 0 | 0 | 1 | 0 | 0 |  | 43F | 0 | 0 | 0 | 0 | 1 | 0 |
| 34I | 0 | 0 | 0 | 1 | 0 | 0 |  | 43K | 0 | 0 | 0 | 0 | 1 | 0 |
| 34J | 0 | 0 | 0 | 1 | 2 | 1 |  | 43L | 0 | 0 | 0 | 0 | 1 | 0 |
| 34K | 0 | 0 | 0 | 1 | 0 | 0 |  | 43N | 0 | 0 | 0 | 0 | 1 | 0 |
| 34L | 0 | 0 | 0 | 1 | 0 | 0 |  | 43P | 0 | 0 | 0 | 0 | 1 | 0 |
| 34M | 0 | 0 | 0 | 1 | 0 | 0 |  | 24A | 0 | 0 | 0 | 0 | 0 | 1 |
| 34O | 0 | 0 | 0 | 1 | 0 | 0 |  | 24AA | 0 | 0 | 0 | 0 | 0 | 1 |
| 47A | 0 | 0 | 0 | 1 | 0 | 0 |  | 24AB | 0 | 0 | 0 | 0 | 0 | 1 |
| 47C | 0 | 0 | 0 | 1 | 0 | 0 |  | 24L | 0 | 0 | 0 | 0 | 0 | 1 |
| 47D | 0 | 0 | 0 | 1 | 0 | 0 |  | 24Q | 0 | 0 | 0 | 0 | 0 | 1 |
| 32B | 0 | 0 | 0 | 1 | 0 | 0 |  | 24U | 0 | 0 | 0 | 0 | 0 | 1 |
| 32E | 0 | 0 | 0 | 1 | 0 | 0 |  | 24W | 0 | 0 | 0 | 0 | 0 | 1 |
| 32F | 0 | 0 | 0 | 1 | 0 | 0 |  | 45B | 0 | 0 | 0 | 0 | 0 | 1 |
| NI-A | 0 | 0 | 0 | 1 | 0 | 0 |  | 45C | 0 | 0 | 0 | 0 | 0 | 1 |
| NI-B | 0 | 0 | 0 | 1 | 0 | 0 |  | 45D | 0 | 0 | 0 | 0 | 0 | 1 |
| NI-E | 0 | 0 | 0 | 1 | 0 | 0 |  | 45F | 0 | 0 | 0 | 0 | 0 | 1 |
| NI-F | 0 | 0 | 0 | 1 | 0 | 0 |  | 45H | 0 | 0 | 0 | 0 | 0 | 1 |
| 43A | 0 | 0 | 0 | 0 | 1 | 0 |  | 45T | 0 | 0 | 0 | 0 | 0 | 1 |
| 43C | 0 | 0 | 0 | 0 | 1 | 0 |  | 45U | 0 | 0 | 0 | 0 | 0 | 1 |
| 43D | 0 | 0 | 0 | 0 | 1 | 0 |  | N1 | 0 | 0 | 0 | 0 | 0 | 1 |
| 43F | 0 | 0 | 0 | 0 | 1 | 1 |  | N2 | 0 | 0 | 0 | 0 | 0 | 1 |
| 43I | 0 | 0 | 0 | 0 | 1 | 0 |  | N4 | 0 | 0 | 0 | 0 | 0 | 1 |
| 43J | 0 | 0 | 0 | 0 | 1 | 0 |  | N5 | 0 | 0 | 0 | 0 | 0 | 1 |
| 43K | 0 | 0 | 0 | 0 | 1 | 0 |  | N6 | 0 | 0 | 0 | 0 | 0 | 1 |
| 43N | 0 | 0 | 0 | 0 | 1 | 0 |  | N7 | 0 | 0 | 0 | 0 | 0 | 1 |
| 43O | 0 | 0 | 0 | 0 | 1 | 0 |  |  |  |  |  |  |  |  |
| 43P | 0 | 0 | 0 | 0 | 1 | 0 |  |  |  |  |  |  |  |  |
| 43R | 0 | 0 | 0 | 0 | 1 | 0 |  |  |  |  |  |  |  |  |
| 43S | 0 | 0 | 0 | 0 | 1 | 0 |  |  |  |  |  |  |  |  |
| 24A | 0 | 0 | 0 | 0 | 0 | 1 |  |  |  |  |  |  |  |  |
| 24AA | 0 | 0 | 0 | 0 | 0 | 1 |  |  |  |  |  |  |  |  |
| 24AD | 0 | 0 | 0 | 0 | 0 | 1 |  |  |  |  |  |  |  |  |
| 24B | 0 | 0 | 0 | 0 | 0 | 1 |  |  |  |  |  |  |  |  |
| 24I | 0 | 0 | 0 | 0 | 0 | 1 |  |  |  |  |  |  |  |  |
| 24K | 0 | 0 | 0 | 0 | 0 | 1 |  |  |  |  |  |  |  |  |
| 24N | 0 | 0 | 0 | 0 | 0 | 1 |  |  |  |  |  |  |  |  |
| 24W | 0 | 0 | 0 | 0 | 0 | 1 |  |  |  |  |  |  |  |  |
| 45A | 0 | 0 | 0 | 0 | 0 | 1 |  |  |  |  |  |  |  |  |
| 45C | 0 | 0 | 0 | 0 | 0 | 1 |  |  |  |  |  |  |  |  |
| 45E | 0 | 0 | 0 | 0 | 0 | 1 |  |  |  |  |  |  |  |  |
| 45F | 0 | 0 | 0 | 0 | 0 | 1 |  |  |  |  |  |  |  |  |
| 45G | 0 | 0 | 0 | 0 | 0 | 1 |  |  |  |  |  |  |  |  |
| 45H | 0 | 0 | 0 | 0 | 0 | 1 |  |  |  |  |  |  |  |  |
| 45K | 0 | 0 | 0 | 0 | 0 | 1 |  |  |  |  |  |  |  |  |
| 45L | 0 | 0 | 0 | 0 | 0 | 1 |  |  |  |  |  |  |  |  |
| 45N | 0 | 0 | 0 | 0 | 0 | 1 |  |  |  |  |  |  |  |  |
| 45R | 0 | 0 | 0 | 0 | 0 | 1 |  |  |  |  |  |  |  |  |
| 45T | 0 | 0 | 0 | 0 | 0 | 1 |  |  |  |  |  |  |  |  |
| N2 | 0 | 0 | 0 | 0 | 0 | 1 |  |  |  |  |  |  |  |  |
| N4 | 0 | 0 | 0 | 0 | 0 | 1 |  |  |  |  |  |  |  |  |
| N7 | 0 | 0 | 0 | 0 | 0 | 1 |  |  |  |  |  |  |  |  |
| N8 | 0 | 0 | 0 | 0 | 0 | 1 |  |  |  |  |  |  |  |  |

ID: Code of the each sample.^+^Population codes. *Sample size.

**Table J. The results of the saturation test for *COI* and C*ytB* sequences.** The results suggest only little saturation for both genes while assuming both symmetrical and asymmetrical topology as significant difference was observed between *Iss* and *Iss*.c values, and *Iss* < *Iss*.*c*.

| **Gene** | **# of OTUs** | ***Iss*** | ***Iss.cSym*** | ***T*** | ***DF*** | ***P*** | ***Iss.cAsym*** | ***T*** | ***DF*** | ***P*** |
| --- | --- | --- | --- | --- | --- | --- | --- | --- | --- | --- |
| ***COI*** | 4 | 0.027 | 0.825 | 75.639 | 242 | 0.0000 | 0.793 | 72.612 | 242 | 0.0000 |
|  | 8 | 0.028 | 0.796 | 73.300 | 242 | 0.0000 | 0.692 | 63.374 | 242 | 0.0000 |
|  | 16 | 0.031 | 0.779 | 75.979 | 242 | 0.0000 | 0.586 | 56.391 | 242 | 0.0000 |
|  | 32 | 0.033 | 0.758 | 78.971 | 242 | 0.0000 | 0.46 | 46.559 | 242 | 0.0000 |
| ***cytB*** | 4 | 0.035 | 0.822 | 55.039 | 164 | 0.0000 | 0.790 | 52.802 | 164 | 0.0000 |
|  | 8 | 0.037 | 0.791 | 52.580 | 164 | 0.0000 | 0.686 | 45.266 | 164 | 0.0000 |
|  | 16 | 0.037 | 0.775 | 55.479 | 164 | 0.0000 | 0.579 | 40.741 | 164 | 0.0000 |
|  | 32 | 0.040 | 0.752 | 57.984 | 164 | 0.0000 | 0.450 | 33.365 | 164 | 0.0000 |

Iss: the index of substitution saturation, *Iss.c*: critical *Iss*, Sym: assumes a symmetrical topology, Asym is *Iss.c* assumes an asymmetrical topology.

| 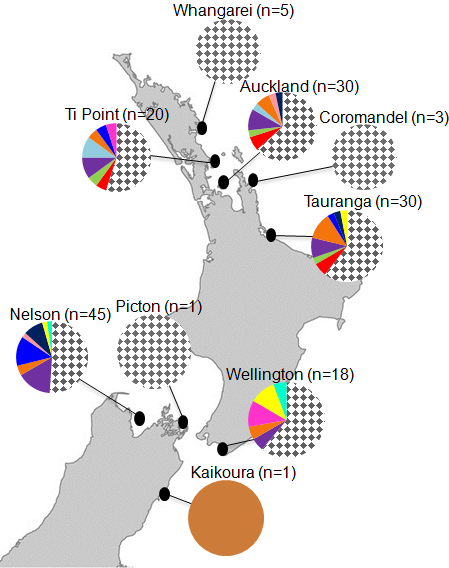  **Figure D. The graphical representation of haplotypes frequencies for *COI* data at each sampling location.** The pie segment represents the relative frequencies of the haplotypes. Each colour corresponds to a different haplotype. Private haplotypes specific to each location are represented with a patterned segment. |  |
| --- | --- |
| **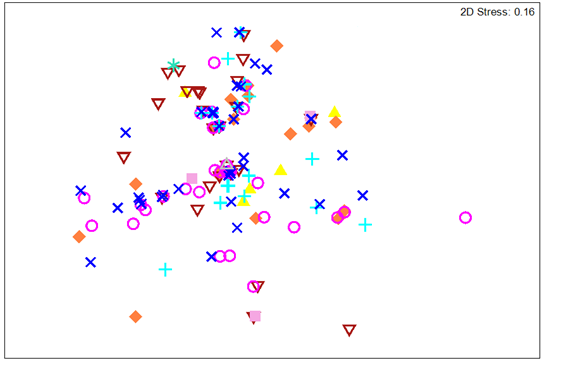**  **Figure E. Non-metric MDS ordination of distances obtained from Cytb sequences.** | |
| **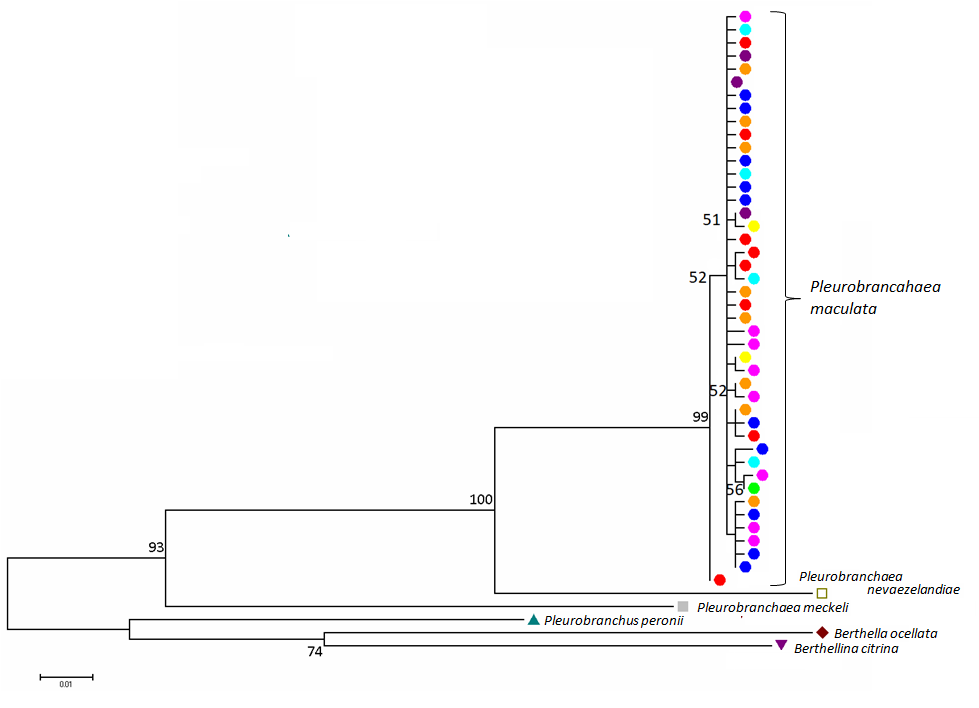 Figure F. Molecular phylogenetic analysis of *COI* sequences by maximum likelihood method.** The tree with the highest log likelihood (-2685.86) is displayed. Initial tree(s) for the heuristic search were obtained automatically by applying Neighbor-Join and BioNJ algorithms to a matrix of pairwise distances estimated using the Maximum Composite Likelihood approach, and then selecting the topology with superior log likelihood value. The analysis involved 49 nucleotide sequences out of a total of 616. The nodes are coloured based on the sampling locations. The numbers on the nodes represent the % bootstrap support (values <50% not presented). | |

**Table K. Detection of first generation migrants.** Likelihood ratio: L_home/L_max. The number of individuals with a probability below the threshold value is 4. The potential migrants are labelled in red (*P* < 0.01) and the most likely population in green.

| ID | Assigned | =-LOG  (L_H_ /L_max_) | Probability | North  -log(L) | South  -log(L) |
| --- | --- | --- | --- | --- | --- |
| 37A | North | 0.000 | 0.5078 | 11.304 | 17.844 |
| 37B | North | 0.000 | 0.5047 | 15.817 | 21.812 |
| 37C | North | 0.000 | 0.5079 | 11.623 | 15.385 |
| 37D | North | 0.000 | 0.5057 | 12.032 | 14.983 |
| 37E | North | 0.000 | 0.5065 | 12.665 | 19.189 |
| 37F | North | 0.000 | 0.5061 | 13.019 | 17.783 |
| 37G | North | 0.000 | 0.5059 | 9.740 | 13.504 |
| 37H | North | 0.000 | 0.5044 | 11.117 | 13.957 |
| 38A | North | 0.000 | 0.5063 | 12.555 | 14.635 |
| 38B | North | 0.000 | 0.5041 | 12.318 | 19.852 |
| 38C | North | 0.000 | 0.5049 | 12.126 | 17.981 |
| 38D | North | 0.000 | 0.5059 | 11.733 | 18.497 |
| 38E | North | 0.000 | 0.5036 | 14.262 | 16.051 |
| 38F | North | 0.000 | 0.5068 | 16.847 | 20.017 |
| 38G | North | 0.000 | 0.5028 | 12.940 | 18.762 |
| 38H | North | 0.000 | 0.5052 | 15.182 | 20.010 |
| 38I | North | 0.000 | 0.5037 | 11.700 | 20.220 |
| 38J | North | 0.000 | 0.5042 | 14.513 | 18.101 |
| 38K | North | 0.000 | 0.5031 | 11.911 | 15.813 |
| 38L | North | 0.000 | 0.5022 | 12.091 | 17.320 |
| NN1 | North | 0.000 | 0.5027 | 12.681 | 17.656 |
| 33A | North | 0.000 | 0.5019 | 12.243 | 17.115 |
| 33B | North | 0.000 | 0.5031 | 12.639 | 18.321 |
| 33C | North | 0.000 | 0.5019 | 13.334 | 17.638 |
| 33D | North | 0.000 | 0.5030 | 11.325 | 17.359 |
| 33E | North | 0.000 | 0.5016 | 17.929 | 25.099 |
| 40A | North | 0.000 | 0.5032 | 9.440 | 18.476 |
| 40B | North | 0.000 | 0.5014 | 14.961 | 20.579 |
| 36A | North | 0.000 | 0.5025 | 10.703 | 15.504 |
| 36B | North | 0.000 | 0.5025 | 10.743 | 17.074 |
| 36C | North | 0.000 | 0.5025 | 10.952 | 15.735 |
| 41A | North | 0.000 | 0.5030 | 13.684 | 14.789 |
| 41B | North | 0.000 | 0.5038 | 9.700 | 15.559 |
| 41C | North | 0.000 | 0.5051 | 13.278 | 18.488 |
| 41D | North | 0.000 | 0.5036 | 11.683 | 19.953 |
| 41E | North | 0.000 | 0.5064 | 12.575 | 21.833 |
| 41F | North | 0.000 | 0.5033 | 13.186 | 16.888 |
| 41G | North | 0.000 | 0.5065 | 13.060 | 20.398 |
| 41H | North | 0.000 | 0.5037 | 11.004 | 16.717 |
| 42A | North | 0.000 | 0.5065 | 11.695 | 19.588 |
| 42B | North | 0.000 | 0.5024 | 15.209 | 16.439 |
| 42C | North | 0.000 | 0.5038 | 11.492 | 19.060 |
| 42D | North | 0.000 | 0.5052 | 11.258 | 15.559 |
| 42E | North | 0.000 | 0.5040 | 14.710 | 21.045 |
| 42F | North | 0.000 | 0.5043 | 10.589 | 13.049 |
| 42G | North | 0.000 | 0.5050 | 11.697 | 20.026 |
| 44A | North | 0.000 | 0.5059 | 10.921 | 13.364 |
| 44B | North | 0.000 | 0.5056 | 12.533 | 15.373 |
| 44C | North | 0.000 | 0.5052 | 12.859 | 19.689 |
| 44D | North | 0.000 | 0.5047 | 12.759 | 13.176 |
| 34A | North | 0.000 | 0.5048 | 11.452 | 12.910 |
| 34B | North | 0.000 | 0.5070 | 11.424 | 20.562 |
| 34C | North | 0.000 | 0.5046 | 14.742 | 16.380 |
| 34D | North | 0.000 | 0.5075 | 10.843 | 14.536 |
| 34E | North | 0.000 | 0.5049 | 9.261 | 14.185 |
| 34F | North | 0.000 | 0.5066 | 17.061 | 19.786 |
| 34G | North | 0.520 | 0.0027 | 15.341 | 14.821 |
| 34H | North | 0.000 | 0.5062 | 13.754 | 18.055 |
| 34I | North | 0.000 | 0.5056 | 11.570 | 15.616 |
| 34J | North | 0.000 | 0.5061 | 13.431 | 16.692 |
| 34K | North | 0.000 | 0.5054 | 10.571 | 17.870 |
| 34L | North | 0.000 | 0.5061 | 11.241 | 15.802 |
| 34M | North | 0.000 | 0.5068 | 11.114 | 12.382 |
| 34N | North | 0.000 | 0.5056 | 12.679 | 16.773 |
| 34O | North | 0.000 | 0.5061 | 12.845 | 17.617 |
| 47A | North | 0.000 | 0.5056 | 12.507 | 17.656 |
| 47B | North | 0.000 | 0.5057 | 15.681 | 17.685 |
| 47C | North | 0.000 | 0.5060 | 14.580 | 16.375 |
| 47D | North | 0.000 | 0.5062 | 14.230 | 15.314 |
| 47E | North | 0.000 | 0.5053 | 10.664 | 17.802 |
| 47F | North | 0.000 | 0.5075 | 11.329 | 18.407 |
| NIA | North | 0.000 | 0.5060 | 9.361 | 14.713 |
| NIB | North | 0.000 | 0.5067 | 11.890 | 19.213 |
| NIC | North | 0.000 | 0.5053 | 9.491 | 13.360 |
| NID | North | 0.000 | 0.5067 | 14.128 | 14.290 |
| NIE | North | 0.000 | 0.5054 | 13.196 | 18.485 |
| NIF | North | 0.000 | 0.5056 | 13.032 | 16.150 |
| NIG | North | 0.000 | 0.5044 | 11.099 | 13.463 |
| 32B | North | 0.000 | 0.5075 | 10.622 | 16.431 |
| 32D | North | 0.000 | 0.5035 | 14.007 | 21.912 |
| 32E | North | 0.000 | 0.5069 | 13.749 | 16.284 |
| 32F | North | 0.000 | 0.5042 | 13.217 | 18.455 |
| 32G | North | 1.541 | 0.0004 | 15.217 | 13.677 |
| 43A | South | 0.000 | 0.5068 | 14.006 | 9.310 |
| 43B | South | 2.303 | 0.0000 | 12.206 | 14.509 |
| 43C | South | 0.149 | 0.0086 | 14.867 | 15.016 |
| 43D | South | 0.000 | 0.5048 | 15.359 | 12.028 |
| 43E | South | 0.000 | 0.5043 | 18.548 | 13.287 |
| 43F | South | 0.000 | 0.5047 | 15.019 | 12.733 |
| 43G | South | 0.000 | 0.5062 | 17.240 | 13.178 |
| 43H | South | 0.000 | 0.5051 | 15.989 | 13.137 |
| 43I | South | 0.000 | 0.5051 | 16.088 | 15.143 |
| 43J | South | 0.000 | 0.5055 | 17.398 | 12.261 |
| 43K | South | 0.000 | 0.5057 | 18.741 | 13.586 |
| 43L | South | 0.000 | 0.5058 | 14.543 | 12.982 |
| 43M | South | 0.000 | 0.5067 | 15.862 | 11.809 |
| 43N | South | 0.000 | 0.5043 | 15.963 | 15.837 |
| 43O | South | 0.000 | 0.5024 | 14.688 | 11.646 |
| 43P | South | 0.000 | 0.5030 | 18.142 | 13.171 |
| 43R | South | 0.000 | 0.5044 | 14.139 | 10.786 |
| 43S | South | 0.000 | 0.5040 | 14.183 | 11.513 |
| 45A | South | 0.000 | 0.5043 | 20.038 | 15.249 |
| 45B | South | 0.000 | 0.5027 | 14.421 | 10.543 |
| 45C | South | 0.000 | 0.5038 | 15.233 | 12.418 |
| 45D | South | 0.000 | 0.5044 | 15.536 | 10.397 |
| 45E | South | 0.000 | 0.5040 | 21.069 | 14.953 |
| 45F | South | 0.000 | 0.5019 | 14.701 | 11.351 |
| 45G | South | 0.000 | 0.5021 | 19.444 | 13.199 |
| 45H | South | 0.000 | 0.5027 | 15.397 | 8.782 |
| 45I | South | 0.000 | 0.5020 | 16.522 | 12.434 |
| 45J | South | 0.000 | 0.5016 | 17.351 | 11.166 |
| 45K | South | 0.000 | 0.5016 | 17.913 | 9.561 |
| 45L | South | 0.000 | 0.5022 | 16.952 | 14.757 |
| 45M | South | 0.000 | 0.5024 | 14.785 | 10.227 |
| 45N | South | 0.000 | 0.5033 | 15.685 | 11.275 |
| 45O | South | 0.000 | 0.5041 | 15.147 | 11.250 |
| 45P | South | 0.000 | 0.5040 | 16.692 | 14.212 |
| 45R | South | 0.000 | 0.5041 | 19.018 | 14.727 |
| 45S | South | 0.000 | 0.5029 | 15.587 | 13.150 |
| 45T | South | 0.000 | 0.5038 | 20.494 | 11.243 |
| 45U | South | 0.000 | 0.5034 | 17.365 | 14.593 |
| 24A | South | 0.000 | 0.5025 | 14.888 | 11.585 |
| 24AA | South | 0.000 | 0.5021 | 16.784 | 10.952 |
| 24AB | South | 0.000 | 0.5010 | 14.717 | 10.333 |
| 24AD | South | 0.000 | 0.5013 | 19.908 | 14.097 |
| 24B | South | 0.000 | 0.5021 | 16.497 | 11.393 |
| 24G | South | 0.000 | 0.5014 | 18.118 | 9.871 |
| 24I | South | 0.000 | 0.5020 | 13.806 | 9.398 |
| 24K | South | 0.000 | 0.5019 | 17.477 | 13.642 |
| 24L | South | 0.000 | 0.5026 | 15.476 | 10.493 |
| 24M | South | 0.000 | 0.5042 | 17.764 | 13.172 |
| 24N | South | 0.000 | 0.5044 | 16.184 | 11.925 |
| 24Q | South | 0.000 | 0.5059 | 17.826 | 14.423 |
| 24U | South | 0.000 | 0.5065 | 16.983 | 8.997 |
| 24V | South | 0.000 | 0.5064 | 15.352 | 12.004 |
| 24W | South | 0.000 | 0.5048 | 18.741 | 14.664 |
| N1 | South | 0.000 | 0.5067 | 19.378 | 11.023 |
| N2 | South | 0.000 | 0.5056 | 18.399 | 10.089 |
| N3 | South | 0.000 | 0.5078 | 15.023 | 11.290 |
| N4 | South | 0.000 | 0.5040 | 16.735 | 15.839 |
| N5 | South | 0.000 | 0.5054 | 12.885 | 8.366 |
| N6 | South | 0.000 | 0.5119 | 15.360 | 12.842 |
| N7 | South | 0.000 | 0.5129 | 13.314 | 9.675 |
| N8 | South | 0.000 | 0.5061 | 14.776 | 12.553 |
| N9 | South | 0.000 | 0.5062 | 17.146 | 11.569 |
| N10 | South | 0.000 | 0.5076 | 18.002 | 13.092 |

ID: ID of the individuals.

| 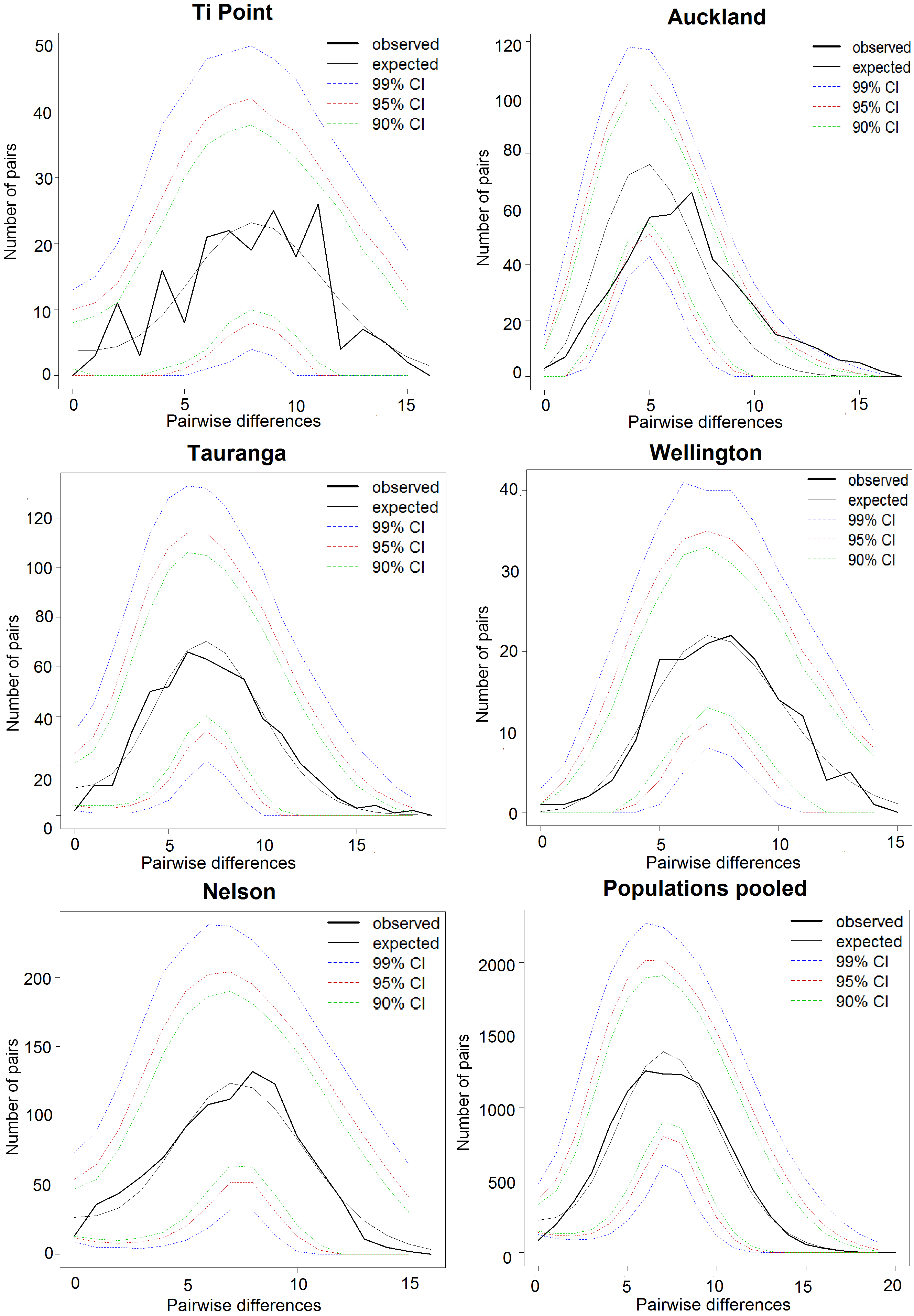    Figure G. Mismatch distribution of pairwise base pair differences between the concatenated *COI* and *CytB* haplotypes. |
| --- |

## References

1. Schuelke M. An economic method for the fluorescent labeling of PCR fragments. Nature Biotechnol. 2000;18(2):233-4.

2. Peakall R, Smouse PE. GENALEX 6: genetic analysis in Excel. Population genetic software for teaching and research. Mol Ecol Notes. 2006;6(1):288-95.

3. Lischer H, Excoffier L. PGDSpider: an automated data conversion tool for connecting population genetics and genomics programs. Bioinformatics. 2012;28(2):298-9.

4. Medina M, Walsh PJ. Molecular systematics of the order anaspidea based on mitochondrial DNA sequence (12S, 16S, and COI). Mol Phylogenet Evol. 2000;15(1):41-58.

5. Wood SA, Taylor DI, McNabb P, Walker J, Adamson J, Cary SC. Tetrodotoxin concentrations in *Pleurobranchaea maculata*: temporal, spatial and individual variability from New Zealand populations. Mar Drugs. 2012;10(1):163-76.

6. Folmer O, Black M, Hoeh W, Lutz R, Vrijenhoek R. DNA primers for amplification of mitochondrial cytochrome c oxidase subunit I from diverse metazoan invertebrates. Mol Mar Biol Biotechnol. 1994;3(5):294-9.

7. Palumbi S. Simple fool's guide to PCR. Privately published. 1991.

8. Merritt T, Shi L, Chase M, Rex M, Etter R, Quattro J. Universal cytochrome b primers facilitate intraspecific studies in molluscan taxa. Mol Mar Biol Biotechn. 1998;7:7-11.

9. Ryman N, Palm S. POWSIM: a computer program for assessing statistical power when testing for genetic differentiation. Mol Ecol Notes. 2006;6(3):600-2.

10. Pritchard JK, Stephens M, Donnelly P. Inference of population structure using multilocus genotype data. Genetics. 2000;155(2):945-59.

11. Weir BS, Cockerham CC. Estimating F-statistics for the analysis of population structure. Evolution. 1984:1358-70.
